# Supplementary material for: Heme allocation in eukaryotic cells relies on mitochondrial heme export through FLVCR1b to cytosolic GAPDH
Source: Nat Commun. 2025 Aug 26;16:7972. doi: 10.1038/s41467-025-62819-2 (PMC12381273; doi:10.1038/s41467-025-62819-2)

Supplementary Data

Heme allocation in eukaryotic cells relies on mitochondrial heme export through FLVCR1b to cytosolic GAPDH

Dhanya Thamaraparambil Jayaram[1], Pranav Sivaram[1], Pranjal Biswas[1], Yue Dai[1], Elizabeth Sweeny[2], and Dennis J. Stuehr[1]

Table S1. Heme content of HEK293T cell supernatants. Heme content was measured by the Oxalic acid fluorometric method on cell supernatants prepared from HEK293T cells that had been treated with either control or siFLVCR1b. The fluorescence emission was measured at 662 nm (excitation 400 nm) relative to standard curves generated with freshly-prepared heme solutions. Values are the mean +/- SD from 3 independent experiments.

Control (pmol/mg protein)	siFLVCR1b (pmol/mg protein)
65 ± 5	60 ± 4

Table S2: Kinetics of heme release from mitochondria into solution. Mitochondria containing ^{14}C-heme were incubated for the indicated times with nothing (control), GAPDH, or GST. Mitochondria were removed by centrifugation prior to measuring heme in the solutions using the spectrophotometric hemochromogen assay. Values are the mean +/- SD from 3 independent experiments.

Time, min	Control, pmol	GAPDH, pmol	GST, pmol
0	684 ± 39	740 ± 32	692 ± 30
15	717 ± 43	1025 ± 103	829 ± 16
30	699 ± 43	1288 ± 74	902 ± 30
60	700 ± 9	1323 ± 104	889 ± 28

Table S3. Heme content of cell supernatants prepared from HEK293T cells treated with either control or siTANGO2. Heme content was measured by the Oxalic acid fluorometric method. The fluorescence emission was measured at 662 nm (excitation 400 nm) relative to standard curves generated with freshly-prepared heme solutions. Values are the mean +/- SD from 3 independent experiments.

Normal (pmol/mg protein)	siTANGO2 (pmol/mg protein)
62 ± 2	66 ± 2

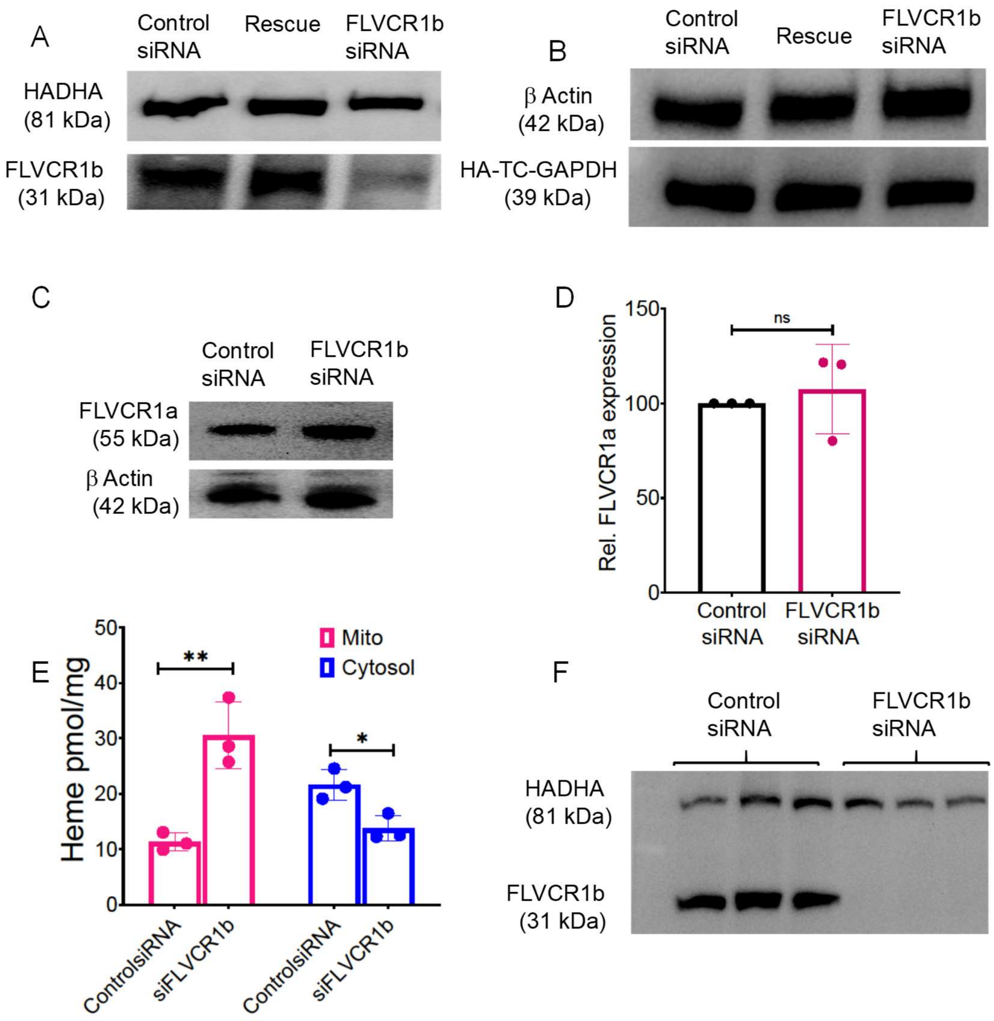

Supplementary Figure 1. Impact of scrambled (control) or FLVCR1b-targeted siRNA treatment on the expression levels of the indicated proteins in transfected HEK293T cells. The samples used were the same as those reported in main Figure 1, and the images are from representative Western blots of three independent trials. Equal total protein amounts of each sample were run on SDS-PAGE and Western blotted and developed using antibodies against the indicated proteins or tags. **A.** HADHA and FLVCR1b expression levels in crude mitochondrial samples from cells that underwent the scrambled or targeted siRNA treatment alone or along with a subsequent transient transfection with FLVCR1b expression plasmid (rescue). **B.** Expression levels of HA-TC-GAPDH and β-Actin proteins in supernatants of cells that received the scrambled or targeted siRNA treatments without or with the subsequent

rescue transfection with FLVCR1b plasmid. **C.** β-Actin and FLVCR1a expression levels in cell cytosols of HEK293T cells that had undergone FLVCR1b knockdown. **D.** Quantitation of the FLVCR1a band intensity from **C** relative to the β-Actin band intensities. From three independent trials, mean +/- SD. Significance: ns vs the compared group based on a two tailed t-test, ns, not significant. t=0.5471, DF=4. **E**. Heme content in mitochondria and cytosol isolated from HEK293T cells in which control siRNA and siFLVCR1b were used. Significance: ** $p < 0.01$ and *$p < 0.05$ vs. groups were compared based on a two tailed t-test, t=5.314, df=4 and t=3.788, df=4 **F**. HADHA and FLVCR1b expression levels in crude mitochondrial samples from cells that underwent the scrambled or targeted siRNA treatment alone. p-values: **D**. Control siRNA vs. siFLVCR1b p=0.6134. **E**. In Mito sample, Control siRNA vs. siFLVCR1b p=0.006 and in Cytosol sample, Control siRNA vs. siFLVCR1b p=0.0193. Abbreviations: HA, Hemagglutinin; Mito, Mitochondria.

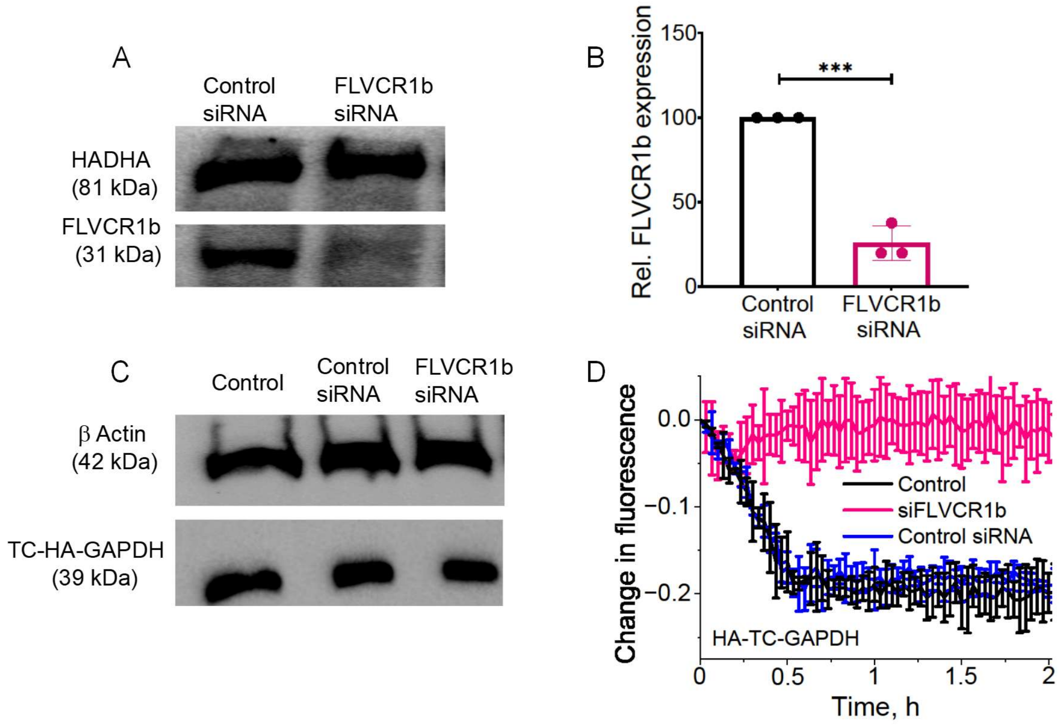

Supplementary Figure 2. Effect of scrambled (control) or FLVCR1b-targeted siRNA treatment on the heme transfer to FlAsH-HA-TC-GAPDH and the expression levels of the indicated proteins in transfected HeLa cells. **A.** Representative Western blot of three independent trials showing HADHA and FLVCR1b expression levels in crude mitochondrial samples from HeLa receiving the indicated treatments. Equal total protein amounts of each sample were run on SDS-PAGE and Western blotted and developed using antibodies against the indicated proteins. **B.** Quantitation of the FLVCR1b band intensity from **A** relative to the HADHA band intensities. From three independent trials, mean +/- SD. Significance: ** $p < 0.01$ vs. the compared group based on a two tailed t-test. t=12.51, DF=4 **C.** Expression levels of HA-TC-GAPDH and β-Actin in supernatants of HeLa cells that had undergone the indicated treatments. Data shown is representative of three trials. **D.** Time course of mitochondrial heme transfer to FlAsH-HA-TC-GAPDH after adding δ-ALA/FeCit in heme depleted medium to HeLa cells that had undergone the indicated treatment. Points are the mean +/- SD of triplicates and the data is representative of three independent trials. p-value: **B**. Control siRNA vs. siFLVCR1b p=0.0002. Abbreviations: HA-TC-, Hemagglutinin-Tetra cysteine-.

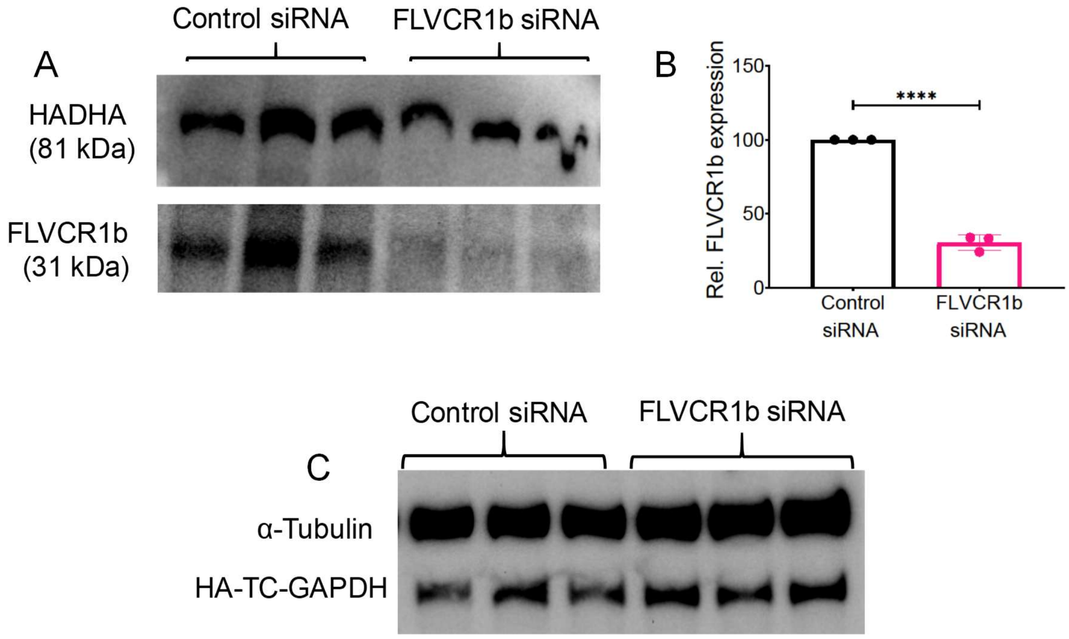

Supplementary Figure S3. Effect of FLVCR1b knockdown on the ability of FlAsH-HA-TC-GAPDH expressed in cells to bind exogenously-supplied versus endogenous heme. HEK293T cells expressing HA-TC-GAPDH underwent a 48 h scrambled (control) or targeted siRNA treatment to knock down FLVCR1b expression, then were given either heme or δ–ALA/Fe and consequent heme binding by FlAsH-HA-TC-GAPDH in the cells was followed by monitoring the fluorescence versus time. **A.** Representative Western blot of three independent trials comparing the levels of HADHA or FLVCR1b expression in crude mitochondrial samples from cells that had undergone the indicated treatments. **B.** Quantitation of the FLVCR1b band intensity from **A** relative to each corresponding HADHA band intensity. From three independent trials, mean mean +/- SD. Significance: **** $p < 0.01$ vs. the compared group based on a two tailed t-test. t=22.32, DF=4 **C.** Western blot indicating the relative expression levels of HA-TC-GAPDH and α-Tubulin in supernatants from the cell groups analysed in panel main figures **1E** and **F** in the text. p-value: Control siRNA vs. siFLVCR1b p=<0.0001. Abbreviations: HA-TC-, Hemagglutinin-Tetra cysteine-.

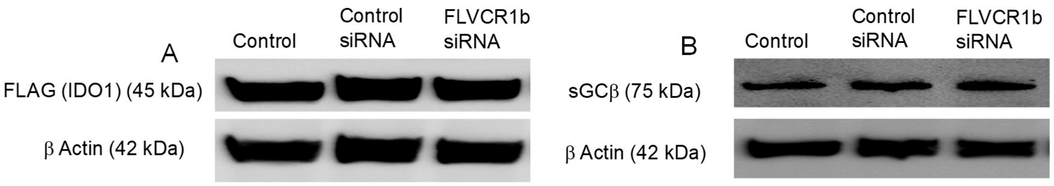

Supplementary Figure S4. Effect of scrambled (control) or FLVCR1b-targeted siRNA treatment on the expression levels of the indicated proteins in HEK293T cell supernatants. **A.** Expression levels of FLAG-IDO1 and β-Actin in supernatants from the cells used in measures of IDO1 activity by the 6 h L-Kynurenine production. **B.** Expression levels of sGCβ and β-Actin in supernatants from the cells used in the measures of mitochondrial heme transfer to FlAsH-labeled TC-sGCβ.

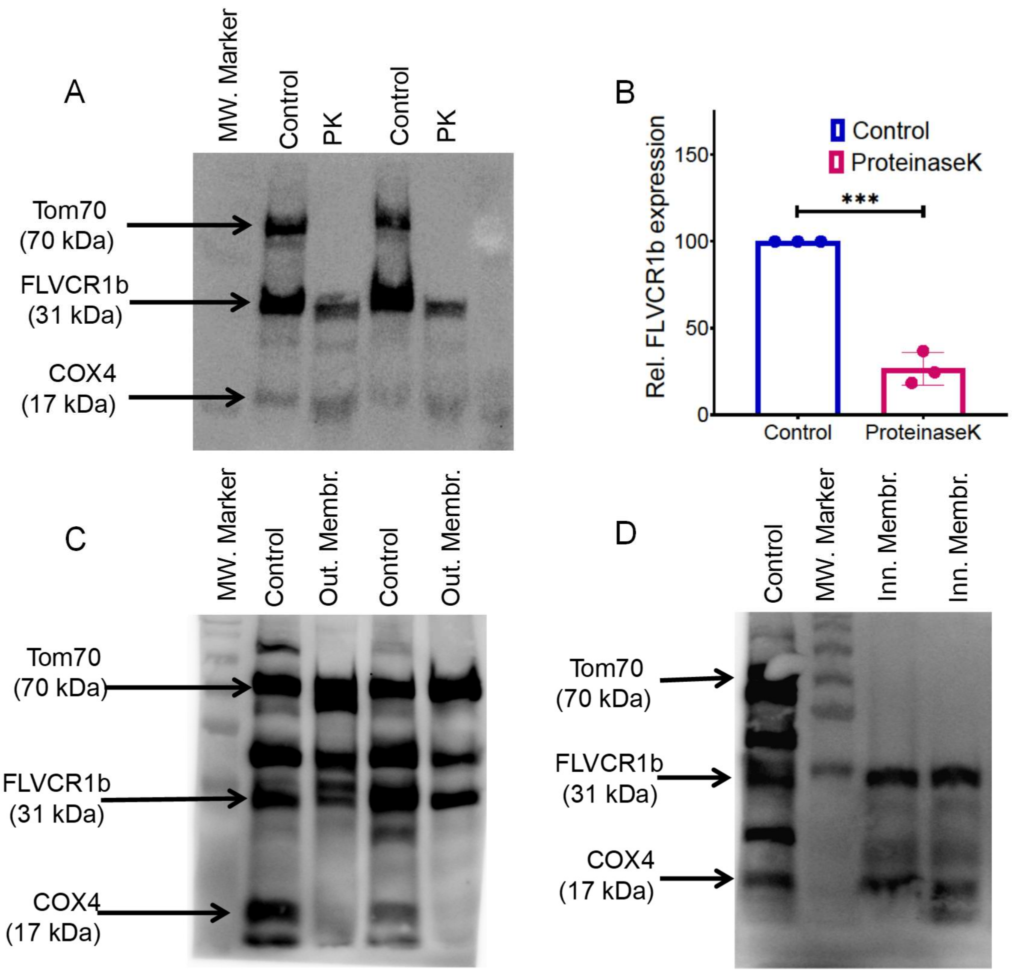

Supplementary Figure S5. Localization of FLVCR1b in the outer and inner mitochondrial membranes. **A.** Representative Western blot of three independent trials of purified mitochondrial samples that did or did not undergo Proteinase K treatment. Proteinase K (5 μg) was added to the resuspended mitochondrial sample (500 μg protein) and then mixed every 5 min for 1 hr at RT before adding 1 μL of 100 mM PMSF. Equal total protein amounts of each sample were run on SDS-PAGE and Western blotted and developed using antibodies against the indicated proteins. **B.** The FLVCR1b band intensity from the Western in A and from two additional independent trials were divided by their respective COX4 band intensities to normalize the FLVCR1b expression. Significance: ***$p < 0.01$ vs. the compared group based on a two tailed t-test. t=13.56, DF=4 **C.** and **D.** Mitochondrial sub-fractionation was done by ultra-centrifugation using a sucrose gradient. The outer and inner membrane samples were collected in different fractions and boiled with 4xLaemmli sample buffer for SDS-PAGE and Western blot analysis using antibodies against the indicated proteins. Data are from two independent trials. p-values: Control siRNA vs. siFLVCR1b p=0.0002. Abbreviations: PK, Proteinase K.

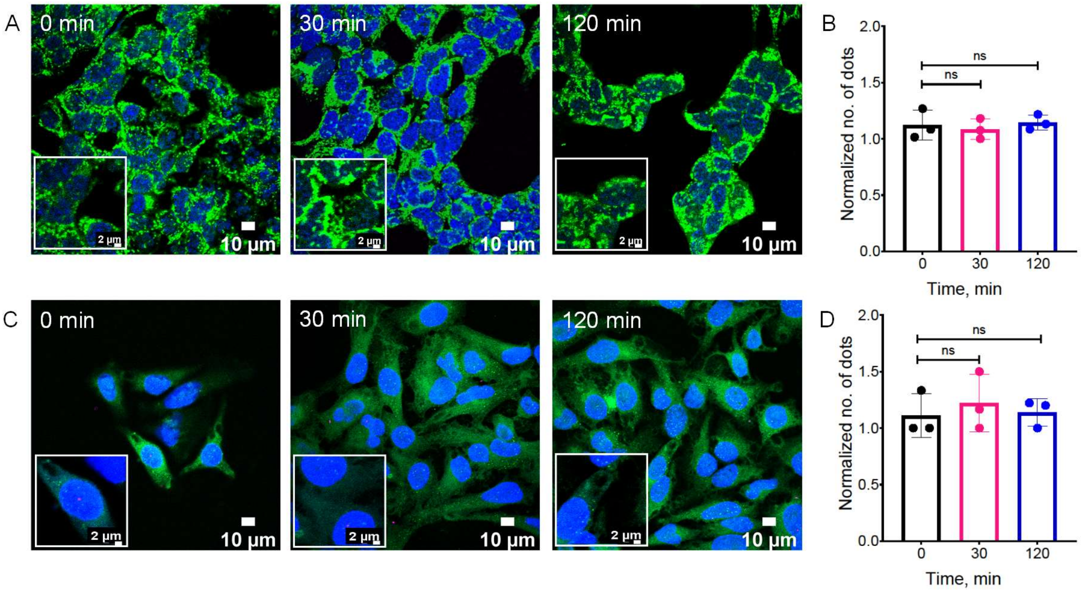

Supplementary Figure S6. Background PLA signals generated in HEK293T or HeLa cells using antibodies directed against FLVCR1b and the non-interacting protein lamelin. Cells were given δ-ALA/Fe to stimulate their mitochondrial heme biosynthesis and then cultured for the indicated times prior to harvest. Cells were stained with DAPI for nuclei (blue), HADHA antibody for mitochondria (green), and FLVCR1 and Lamelin antibodies to show their association by PLA (pink). **A**. and **C**. Representative fluorescence microscope images of the HEK293T and HeLa cells, respectively, at 0, 30, and 120 min, n=3. **B.** Quantification of PLA signals in images from **A** and from two additional independent trials. F= 0.2728, DF=6. **D**. Quantification of PLA signals in images from **C** and from two additional independent trials. F= 0.2551, DF=6. Each image was quantified using Volocity 6.5.1 (Quorum Technologies) image analysis software. Significance: ns vs the compared group based on a one-way ANOVA test. ns, not significant. p-values: **B**. 0 vs. 30 p=0.8664, 0 vs. 120 p=0.9446. **D**. 0 vs. 30 p=0.727, 0 vs. 120 p=0.9756.

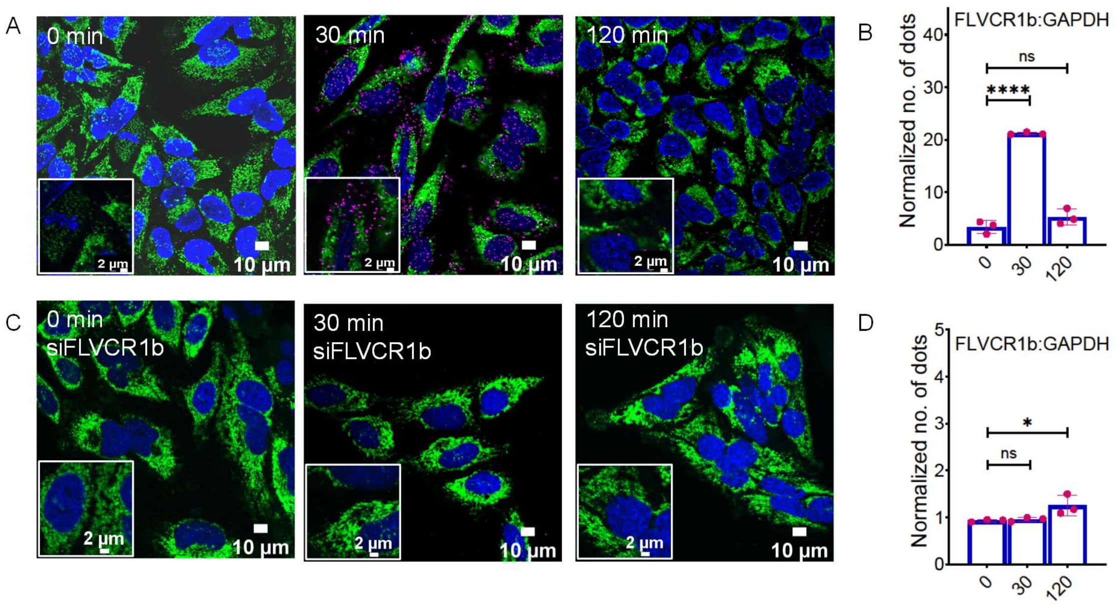

Supplementary Figure S7. GAPDH association with FLVCR1b in HeLa cells at the indicated time points as determined by PLA after giving the cells δ-ALA/Fe to stimulate their mitochondrial heme biosynthesis for the indicated times. After fixation the cells were stained with DAPI for nuclei (blue), HADHA antibody for mitochondria (green), and GAPDH and FLVCR1 antibodies to show their association by PLA (pink). **A and C** Representative fluorescence microscope images of cells that either had not or had undergone FLVCR1b knockdown by targeted siRNA treatment, respectively, n=3. **B** Quantification of PLA signals in images shown in **A** and from two additional independent trials. F=233, DF=6. **D** Quantification of PLA signals in images shown in **C** and from two additional independent trials. F=5.791, DF=6. Mean +/- SD of 3 independent trials. Significance: ns, * $p < 0.05$, and **** $p < 0.0001$ vs. the compared group based on a one-way ANOVA test. ns, not significant. Microscope images were quantified using Volocity 6.5.1 (Quorum Technologies) image analysis software. p-values: **B.** 0 vs. 30 p=<0.0001, 0 vs. 120 p=0.1383. **D**. 0 vs. 30 p=0.945, 0 vs. 120 p=0.0381.

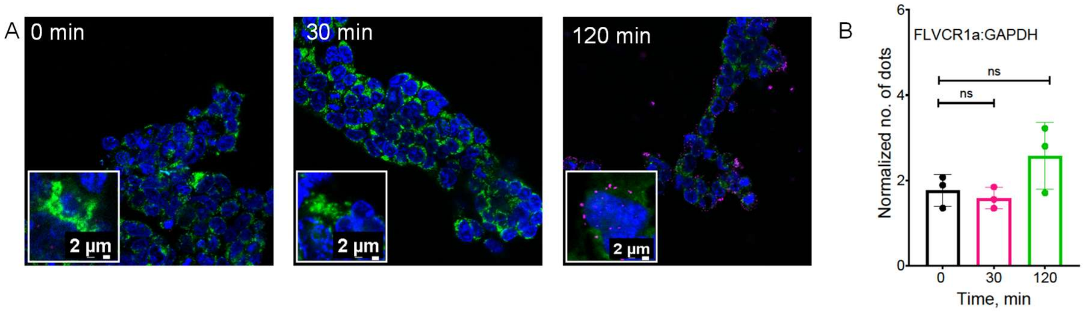

Supplementary Figure S8. GAPDH & FLVCR1a association in HEK293T cells monitored by PLA after giving the cells δ-ALA /Fe to stimulate their mitochondrial heme biosynthesis. Cells were fixed at the indicated time points, and then were stained with DAPI for nuclei (blue), HADHA antibody for mitochondria (green), and GAPDH and FLVCR1a antibodies to show their association by PLA (pink). **A.** Representative fluorescence microscope images of the cells at 0, 30, and 120 min after the addition of δ-ALA /Fe, n=3. **B.** Quantification of PLA signals in images shown in **A** and from two additional independent trials. Microscope images were quantified using Volocity 6.5.1 (Quorum Technologies) image analysis software. Mean +/- SD. Significance: ns, vs. the compared group based on a one-way ANOVA test. ns, not significant. F= 3.078, DF=6. p-values: **B**. 0 vs. 30 p=0.8706, 0 vs. 120 p=0.1793.

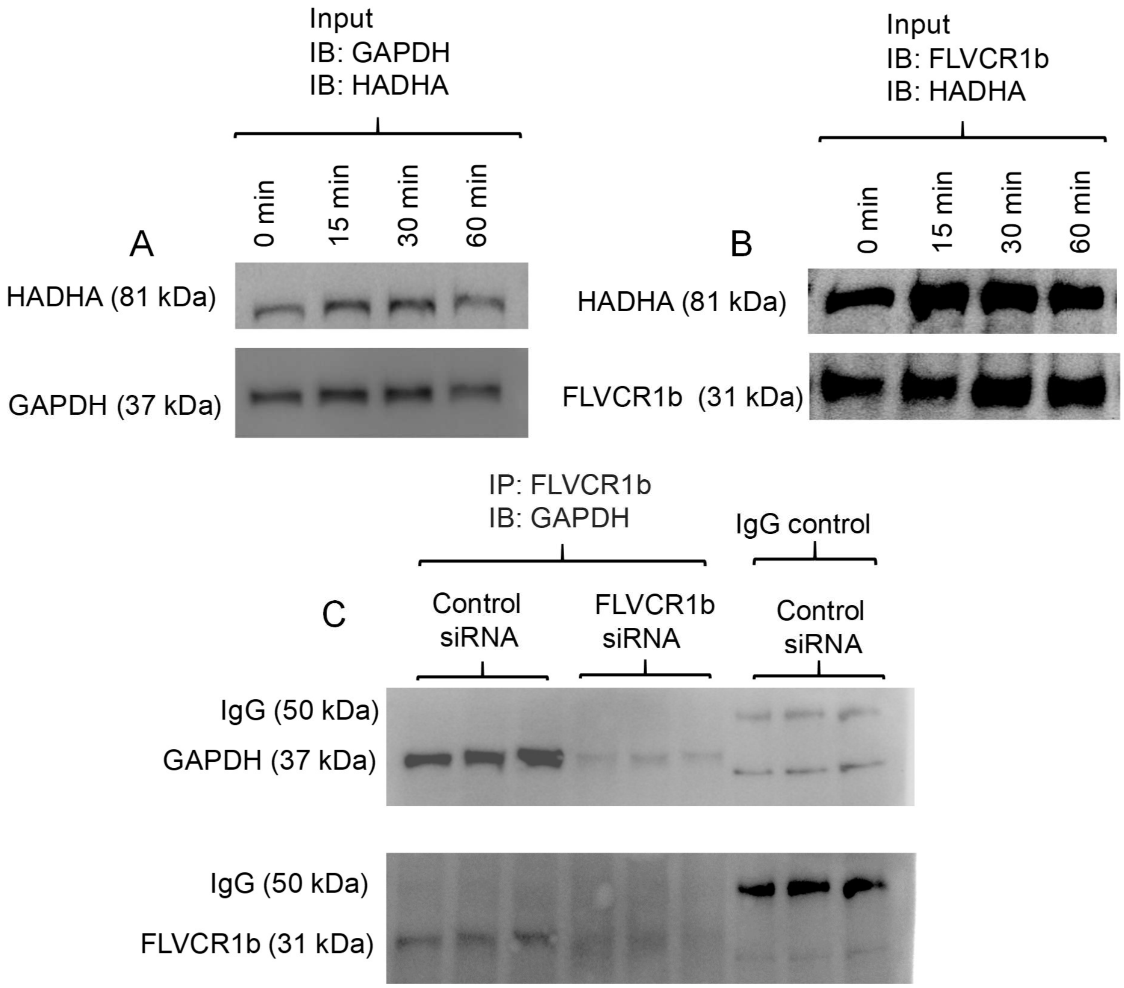

Supplementary Figure S9. **A**. and **B**. Representative western blots showing the input expression levels of HADHA, GAPDH, and FLVCR1b in the crude mitochondrial samples used for the immunoprecipitations in main figures **2C** and **D** in the text. **C,** n=3. Representative western blots showing relative levels of FLVCR1b and GAPDH proteins in the IP samples for the cells that were treated with scrambled (control) or FLVCR1b-targeted siRNA. Abbreviation: IP, Immunoprecipitation. IB, Immunoblot.

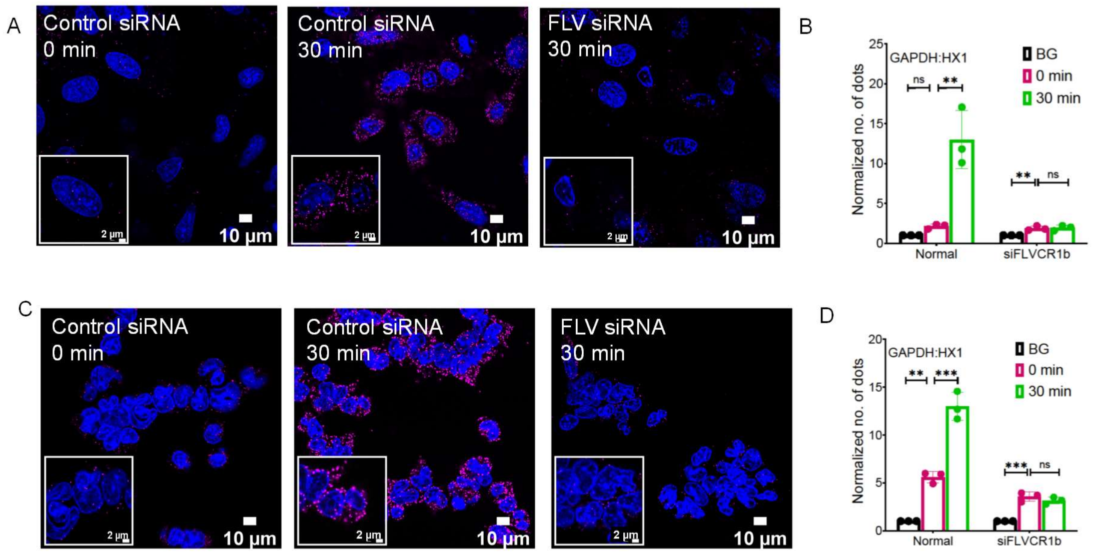

Supplementary Figure S10. Effect of FLVCR1b knockdown on the association of GAPDH with mitochondria in HEK293T and HeLa cells as determined by PLA at time points following δ-ALA /Fe addition to the cells. HEK293T or HeLa cells that had been transfected with scrambled (control) or FLVCR1b-targeted siRNA were given δ-ALA /Fe to stimulate their mitochondrial heme biosynthesis and then were harvested at the indicated times. After fixation the cells were stained with DAPI for nuclei (blue) and antibodies against GAPDH and the outer mitochondrial membrane protein Hexokinase 1 (HX1) to show their association by PLA (pink). **A** and **C**. Representative fluorescence microscope images of HEK293T and HeLa cells respectively at 0, 30 and 120 min, n=3. **B**. and **D**. Quantification of the PLA signals in the cell images of **A** and **C** along with two additional independent trials. BG, background PLA signal. Microscope images were quantified using Volocity 6.5.1 (Quorum Technologies) image analysis software. **B, D.** Mean +/- SD of 3 independent trials. Significance: * $p < 0.05$, ** $p < 0.01$, *** $p < 0.001$, and **** $p < 0.0001$ vs. the compared group based on a one-way ANOVA test. ns, not significant. For **B**. F=29.70, DF=6. And **D**. F= 131.7, DF=6. p-value: **B**. In HEK, control cells, 0 min vs. BG p=0.741, 0 min vs. 30 min p=0.0008. siFLVCR1b cells, 0 min vs. BG p=0.0102, 0 min vs. 30 min p=0.9578. In HeLa, control cells, 0 min vs. BG p= 0.0015, 0 min vs. 30 min p=0.0001. siFLVCR1b cells, 0 min vs. BG p=0.0002, 0 min vs. 30 min p=0.3395. Abbreviations: BG, Background.

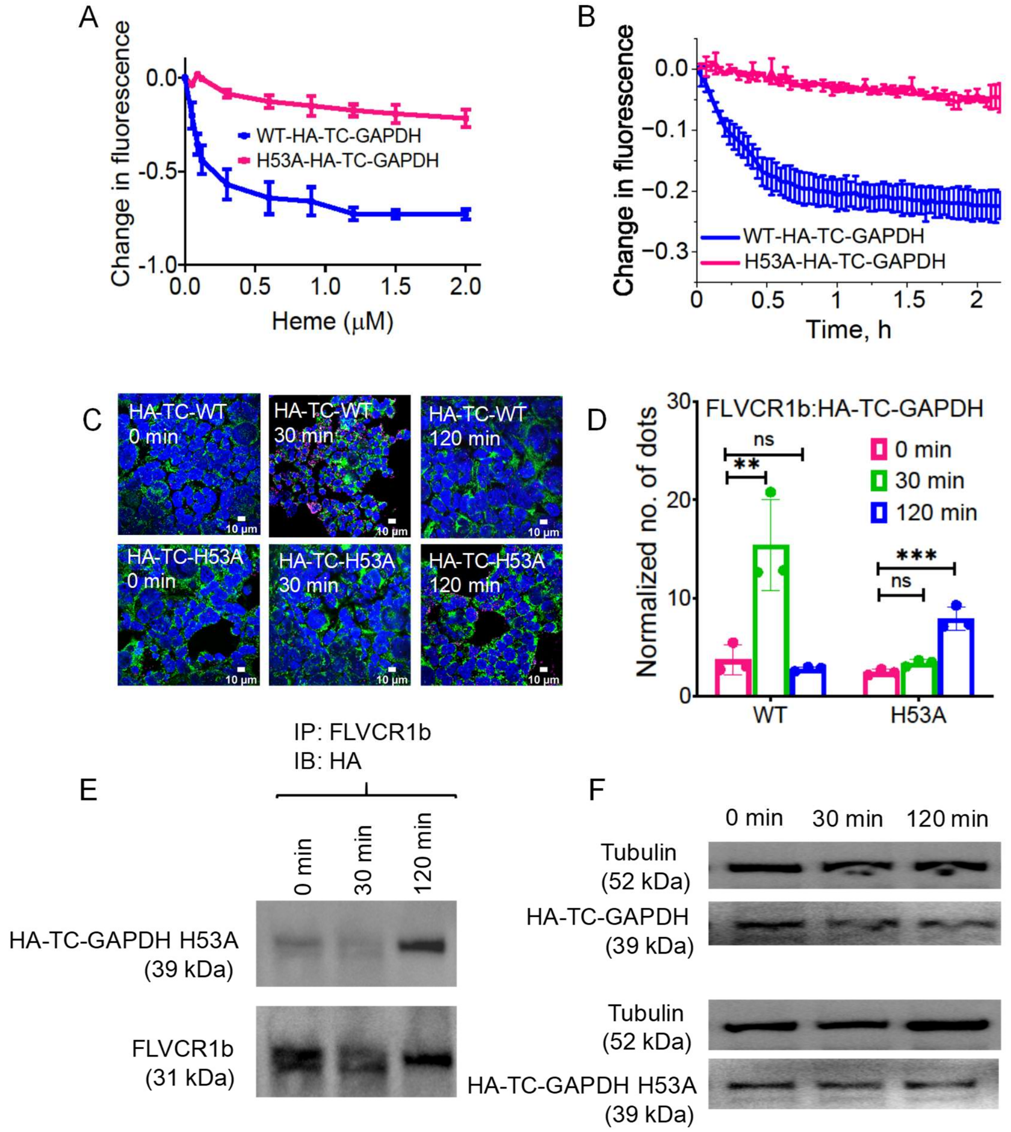

Supplementary Figure S11. A Heme titration of FlAsH-labeled HA-TC-GAPDH (WT and H53A) in cell supernatants (0.08 mg/ml) mixed in buffer (50 mM MOPS, 150 mM NaCl, pH 7.4) at RT with varying concentrations of heme (0–2 μM). The end point fluorescence signals were measured in the 96-well plate reader at RT using excitation at 508 nm and emission at 528 nm. Values are the mean +/- SD from 3 independent experiments. **B** Live cell heme binding/transfer kinetics using FlAsH labeled HA-TC-GAPDH. Change in the cell heme level with time after providing δ-ALA /Fe to the HEK293T cells cultured in a 96-well plate with HA-TC-GAPDH or its H53A variant transfection followed by FlAsH labelling. Values are the mean +/- SD from 3 independent experiments. **C** PLA indicating FLVCR1b association with HA-tagged versions of GAPDH wild type or the heme binding-defective variant H53A GAPDH. **D** Quantification of PLA results showing that the temporal increase in FLVCR1b-HA-TC-GAPDH

association was muted and delayed for the H53A variant that cannot bind heme. **E** HEK293T cells expressing the HA-TC-GAPDH H53A variant were harvested at the indicated time points after δ-ALA /Fe addition and cell supernatant samples (equal total protein) were subject to immunoprecipitation using anti-FLVCR1 antibody. Representative Western blot shows the relative levels of HA-TC-GAPDH H53A and FLVCR1b proteins in the immunoprecipitation samples, n=3. **F** Representative western blots showing the relative expression levels of α-Tubulin, HA-TC-GAPDH, or the HA-TC-GAPDH H53A variant in supernatant samples (equal total protein) from transfected HEK293T cells after they had received δ-ALA /Fe and underwent further culture for the indicated times prior to harvest, n=3. **D** Mean +/- SD of 3 independent trials. Significance: ** $p < 0.01$, *** $p < 0.001$, and **** $p < 0.0001$ vs. the compared group based on a one-way ANOVA test. ns, not significant. p-values: **D**. In FLVCR1b: WT-HA-TC-GAPDH, 0 vs. 30 p=0.0042, 0 vs. 120 p=0.8873. In FLVCR1b:H53a-HA-TC-GAPDH, 0 vs. 30 p=0.2681, 0 vs. 120 p=0.0002. Abbreviation: IP, Immunoprecipitation. IB, Immunoblot. HA-TC-, Hemagglutinin-Tetra cysteine-.

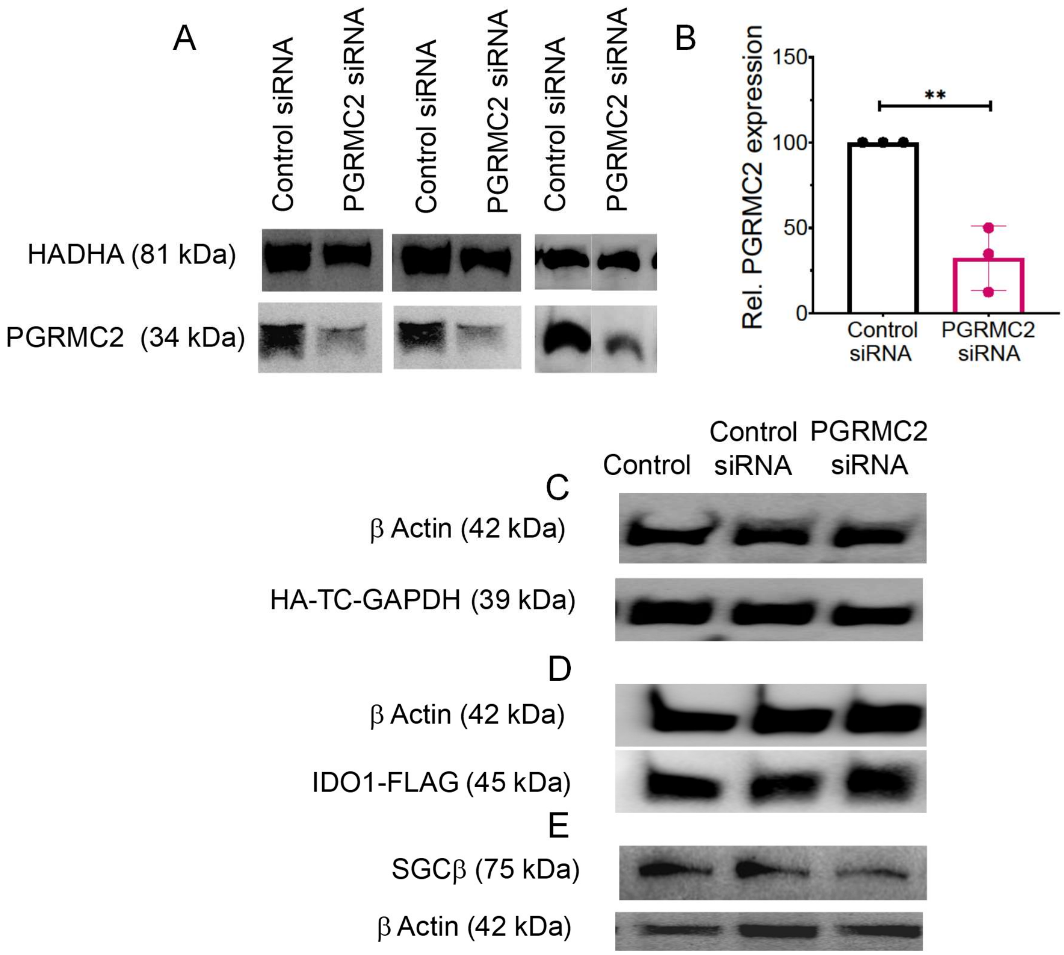

Supplementary Figure S12. Impact of scrambled (control) or PGRMC2-targeted siRNA treatment on the expression levels of the indicated proteins in transfected HEK293T cells. The images are from representative Western blots after running samples containing equal total protein on SDS-PAGE and developing using antibodies against the indicated proteins or tags, n=3. **A**. HADHA and PGRMC2 expression levels in cell crude mitochondrial samples. **B**. Quantitation of the results from **A** based on band intensities. Mean +/- SD. Significance: ** $p < 0.01$ vs. the compared group based on a two tailed t-test. t= 6.190, DF= 4 **C, D, E**. Representative Western blots of three independent trials indicating the expression levels of HA-TC-GAPDH, IDO10FLAG, sGCβ, and β-actin in the supernatants of cells that had been given the vehicle, scrambled siRNA, or PGRMC2-targeted siRNA. p-value: Control siRNA vs. siPGRMC2 p=0.0035. Abbreviation: HA-TC-, Hemagglutinin-Tetra cysteine-.

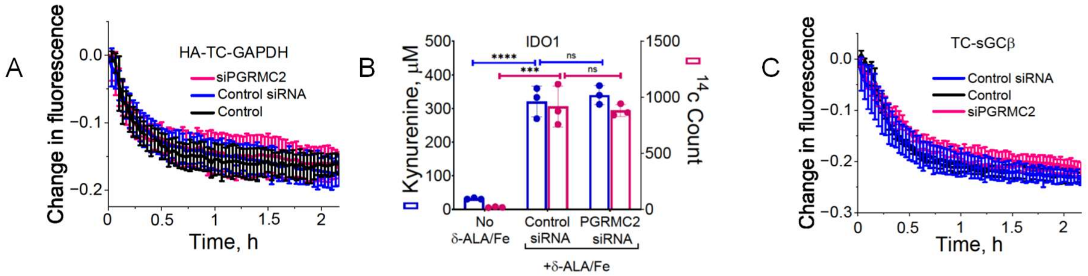

Supplementary Figure S13. Knockdown of cell PGRMC2 protein expression does not impact the transfer of mitochondrial heme into HA-TC-GAPDH or its downstream heme deliveries to IDO1 or sGCβ. HEK293T cells underwent treatment with vehicle alone (control) or with scrambled (control siRNA) or PGRMC2-directed siRNA, and were transfected to express either HA-TC-GAPDH, IDO1, or TC-sGCβ. Cells were then given cold or ^{14}C radiolabeled δ-ALA/Fe and further cultured. **A**. Time course of FlAsH-HA-TC-GAPDH fluorescence change indicating its heme binding, after cell heme biosynthesis was stimulated by addition of δ-ALA /Fe at time = 0. **B**. IDO1 activity in cells after d-ALA/ FeCit addition as assessed by the heme-dependent IDO1 production of L-Kynurenine that had accumulated in the cell culture fluid after 6 h; and by the content of IDO1 ^{14}C-heme, both as measured from IDO1 immunoprecipitation of cell supernatants (equal total protein) made from cells harvested 6 h after addition of ^{14}C-δ-ALA/Fe. **C**. Time course of FlAsH-TC-sGCβ heme binding in live cells after they were given δ-ALA/Fe at Time = 0. **A**, **C** Representative of three independent trials, data points are the mean +/- SD of triplicates. **B** Three independent trials, mean +/- SD. Significance: **** $p < 0.0001$, *** $p < 0.001$ vs. the compared group based on a one-way ANOVA. ns, not significant. F=63.91, DF=6. p-value: In Kynurenin assay, Control siRNA+no Heme vs. Control siRNA+Heme p=<0.0001, Control siRNA+Heme vs. siFLVCR1b+Heme p= 0.6898. In heme count, Control siRNA+no Heme vs. Control siRNA+Heme p=0.0001, Control siRNA+Heme vs. siFLVCR1b+Heme p= 0.9195. Abbreviation: HA-TC-, Hemagglutinin-Tetra cysteine-; δ-ALA/Fe , δ-aminolevulinic acid and ferric citrate; μM, micromolar.

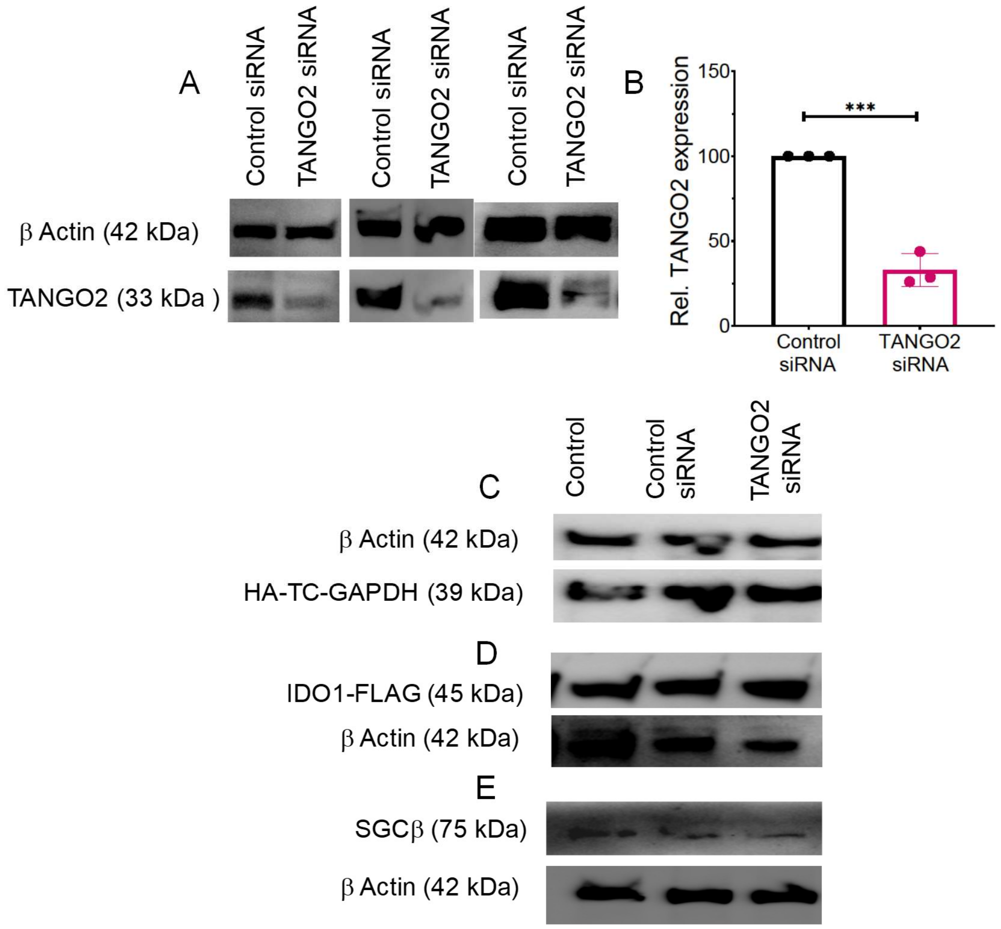

Supplementary Figure S14. Impact of scrambled (control) or TANGO2-targeted siRNA treatment on the expression levels of the indicated proteins in transfected HEK293T cells. The images are from representative Western blots, n=3. Equal total protein amounts of each cell supernatant sample were run on SDS-PAGE and Western blotted and developed using antibodies against the indicated proteins or tags. **A**. β-actin and TANGO2 expression levels in response to the scrambled or targeted siRNA treatment. **B**. Quantitation of the results from **A** based on band intensities. Three independent trials, mean +/- SD. Significance: *** $p < 0.001$ vs. the compared group based on a two-tailed t-test. t= 12.09, DF= 4 **C, D, E**. Representative Western blots of three independent trials indicating the expression levels of HA-TC-GAPDH, IDO1-FLAG, sGCβ, and β-actin in the supernatants of the cells that had been given vehicle, scrambled siRNA, or TANGO2-targeted siRNA. p-value: Control siRNA vs. siTANGO2 p=0.0003. Abbreviation: HA-TC-, Hemagglutinin-Tetra cysteine-.

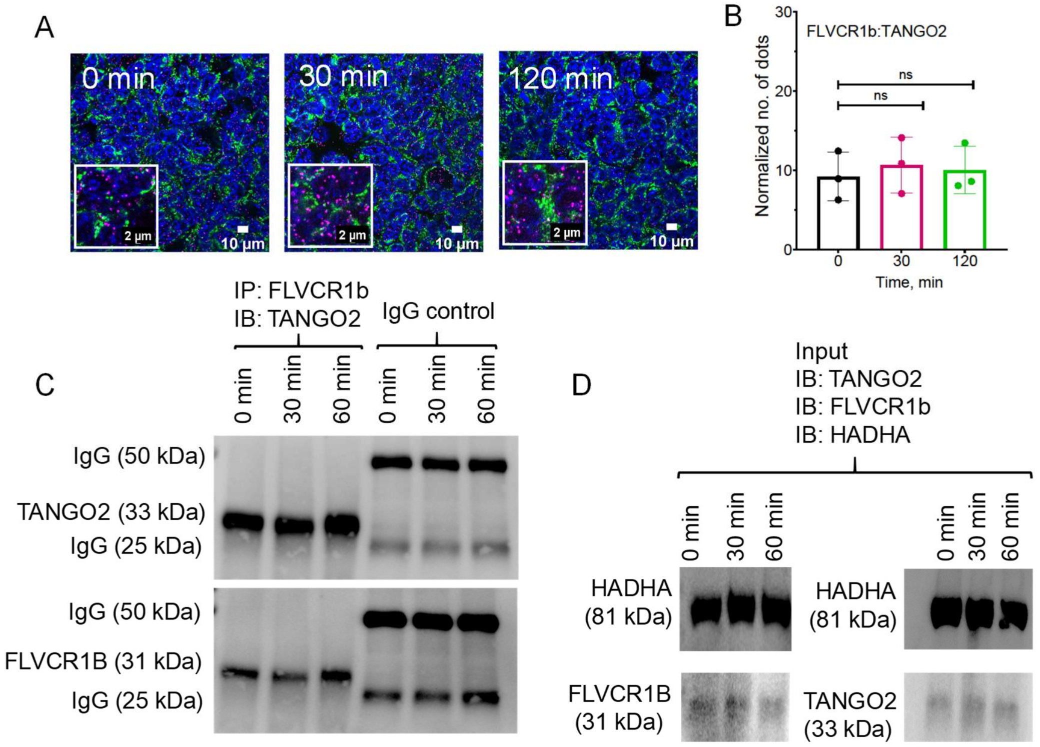

Supplementary Figure S15. TANGO2 interaction with FLVCR1b in cells does not change after stimulation of their mitochondrial heme biosynthesis. **A**. PLA was used to assess TANGO2 association with FLVCR1b. Representative fluorescence microscope images of cells stained with DAPI against nuclei (blue), mitochondria (HADHA antibody, green), and antibodies against TANGO2 and FLVCR1b to indicate their association by PLA (pink). Inset shows zoomed images n=3. **B**. Microscope images were quantified using Volocity 6.5.1 (Quorum Technologies) image analysis software. Normalization of PLA signals was done by subtracting the background PLA signal obtained using antibodies against FLVCR1b and a non-interacting partner protein (laminin). Three independent trials, mean +/- SD. Significance: ns, not significant vs. the compared group based on a one-way ANOVA. F=0.1594, DF=6. **C**. Representative Western blots of three independent trials showing the relative levels of FLVCR1b and TANGO2 expression in crude mitochondrial samples prepared from cells harvested at the indicated times after δALA/Fe addition and subject to immunoprecipitation using an anti-FLVCR1 antibody (equal total protein). Antibody against IgG Isotype control was also used to check for specific and non-specific binding to the FLVCR1b antibody on beads. **D**. Western blots showing the input expression levels of TANGO and FLVCR1b in cell supernatants used for the immunoprecipitation. p-value: 0 vs. 30 p=0.8036, 0 vs. 120 p=0.9303. Abbreviation: IP, Immunoprecipitation. IB, Immunoblot. HA-TC-, Hemagglutinin-Tetra cysteine-.

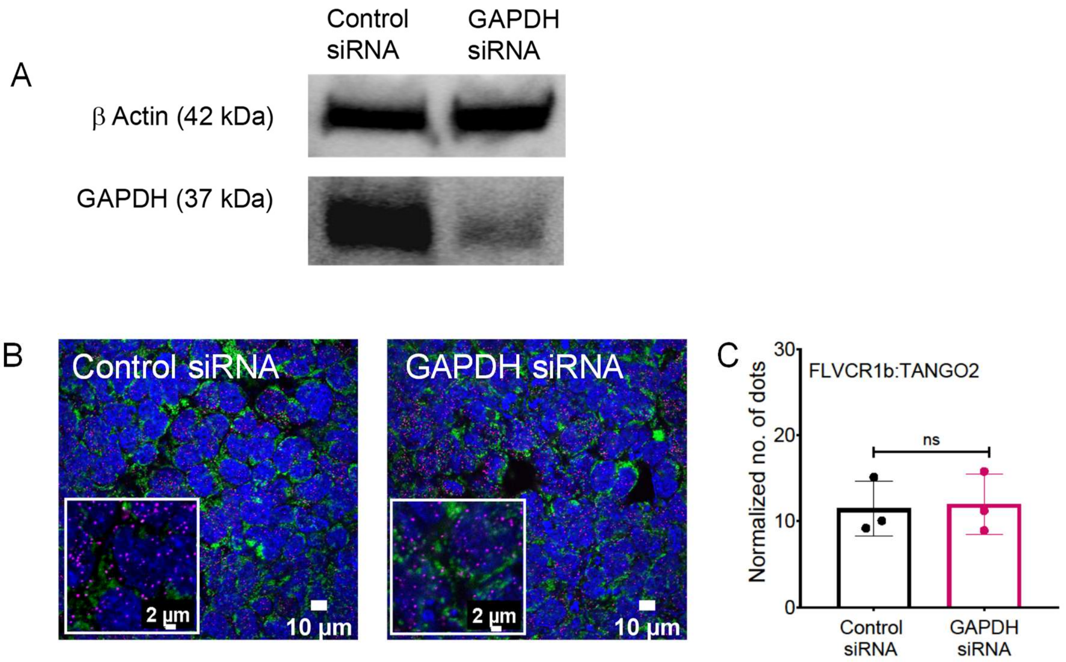

Supplementary Figure S16. Impact of GAPDH knockdown on the TANGO2 association with FLVCR1b in cells. HEK293T cells were treated with scrambled (control) or GAPDH-directed siRNA and then further cultured for 48 h before processing. **A**. Representative Western blot of three independent trials of cell supernatants showing the change in their GAPDH content in response to the siRNA treatments. **B**. PLA was used to assess TANGO2 association with FLVCR1b. Representative fluorescence microscope images of three independent trials of cells stained with DAPI against nuclei (blue), mitochondria (HADHA, green), and to show the TANGO2-FLVCR1b association by PLA (pink). **C.** Microscope images from **A** and two additional independent trials were used to quantify the TANGO2-FLVCR1b association using Volocity 6.5.1 (Quorum Technologies) image analysis software. Normalization of PLA signals was done by subtracting the background PLA signal obtained using antibodies against FLVCR1b and a non-interacting partner protein (laminin). Three independent trials, mean +/- SD. Significance: ns vs. the compared group based on a two-tailed t-test. ns, not significant. t=0.1846, DF=4. p-value: Control siRNA vs. siGAPDH p=0.8625.

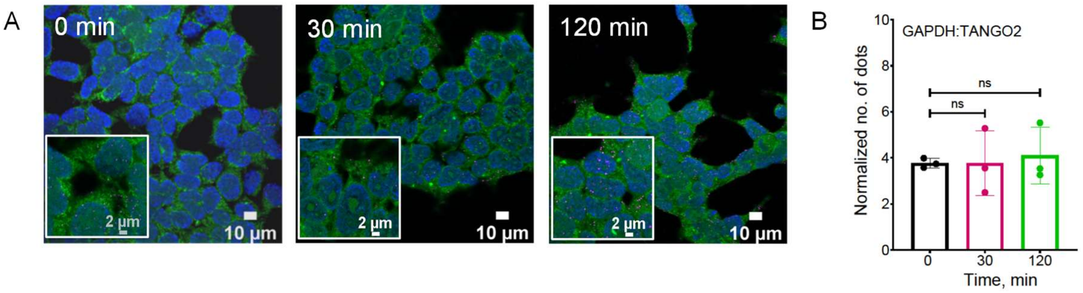

Supplementary Figure 17. GAPDH associates weakly with TANGO2 in cells. HEK293T cells were given δ-ALA /Fe at time = 0 and incubated further for the indicated times before being processed. **A**. Representative fluorescence microscope images of cells stained with DAPI against nuclei (blue), mitochondria (HADHA, green), and to show the TANGO2-GAPDH association by PLA (pink) at the indicated times, n=3. **B**. Quantification of the PLA results using Volocity 6.5.1 (Quorum Technologies) to indicate the level of TANGO2 and GAPDH association versus time during the δ-ALA/Fe incubation. Normalization of PLA signals was done by subtracting the background PLA signal obtained using antibodies against TANGO2 and a non-interacting partner protein (laminin). Three independent trials, mean +/- SD. Significance: one-way ANOVA. ns, not significant. F= 0.09535, DF=6. p-value: 0 vs. 30 p=0.9998, 0 vs. 120 p=0.8991.

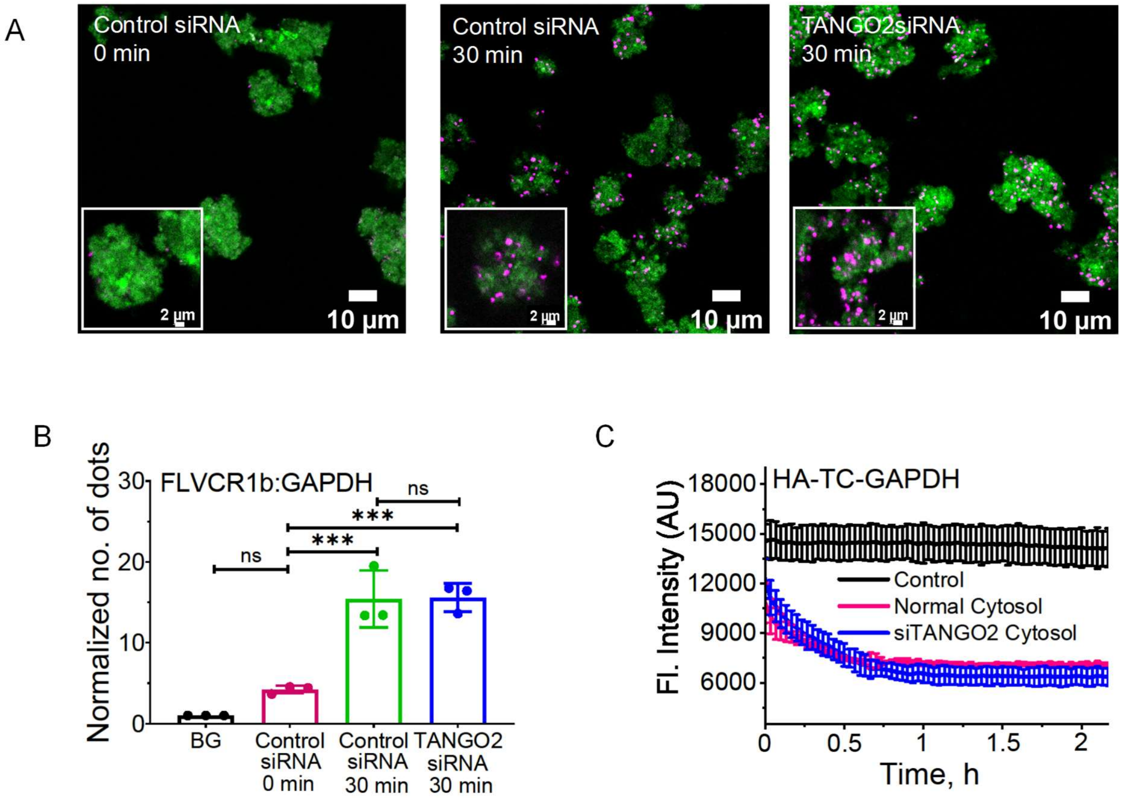

Supplementary Figure 18. Cytosolic TANGO2 is not needed for GAPDH to associate with FLVCR1b in purified mitochondria or for the mitochondria to transfer their heme to GAPDH. **A**. The panels show representative fluorescence microscope images of the mitochondria harvested at the indicated incubation times, after staining with antibodies against mitochondria (HADHA, green) and against GAPDH and FLVCR1b to indicate their level of association by PLA (pink), n=3. **B**. PLA data comparing the relative levels of FLVCR1b and GAPDH association in mitochondria that were reisolated after they had been incubated for the indicated times with δ-ALA, ADP, and mitochondria-free cell supernatants prepared either from control or TANGO2 knockdown HEK293T cells. BG, background PLA signal. F=43.90, DF=8. **C**. Fluorescence traces indicating the kinetics of heme binding by FlAsH-HA-TC-GAPDH after adding buffer alone (control) or buffer containing the reisolated mitochondria that had been incubated with δ-ALA, ADP, and mitochondria-free cell supernatants prepared either from control or TANGO2 knockdown HEK293T cells. P-values: **B**. In ControlsiRNA cells, 0 min vs. 30 minp=0.0031, 0 min vs. 120 min p=0.9712 and in TANGO2siRNA cells, 0 min vs. 30 min p=0.4813, 0 min vs. 120 min p=0.0319 **C**. BG vs. Control p=0.2689, Control 0 min vs. Control 30 min p=0.0005, Control 0 min vs. siTANGO2 30 min p=0.0005, Control 30 min vs. siTANGO2 30 min p=0.9995. Abbreviation: HA-TC-, Hemagglutinin-Tetra cysteine-.

Full western blot scan for Supplementary Figure 1A

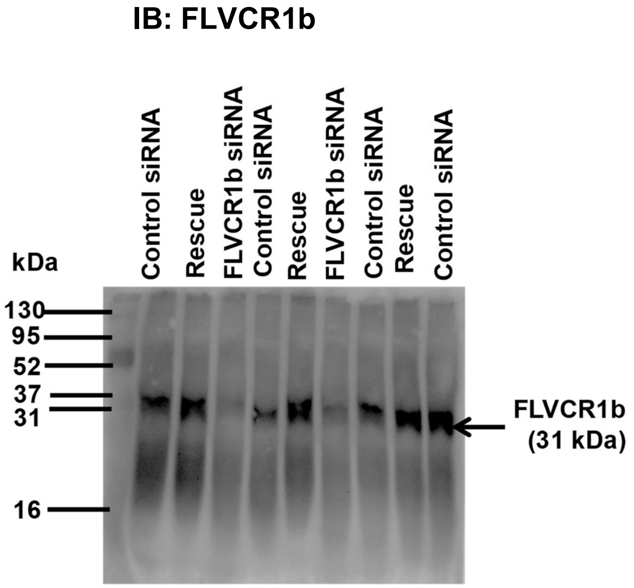

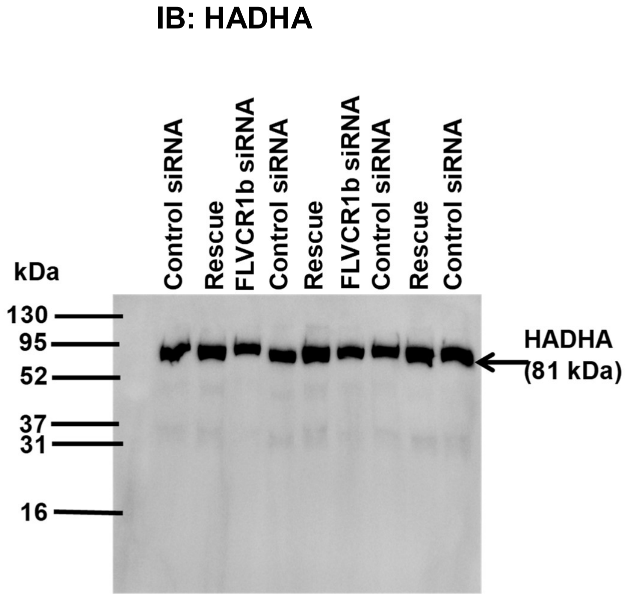

Full western blot scan for Supplementary Figure 1B

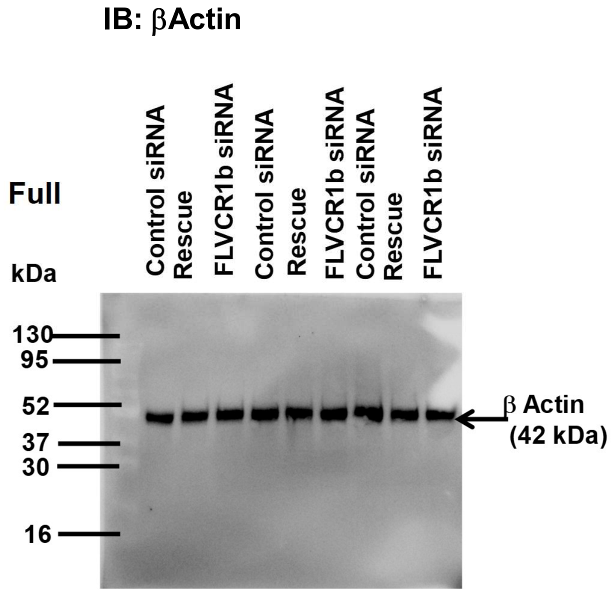

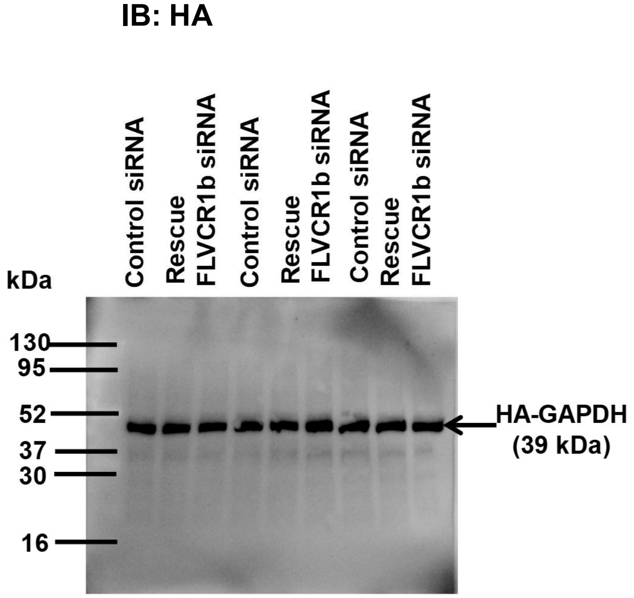

Full western blot scan for Supplementary Figure 1C

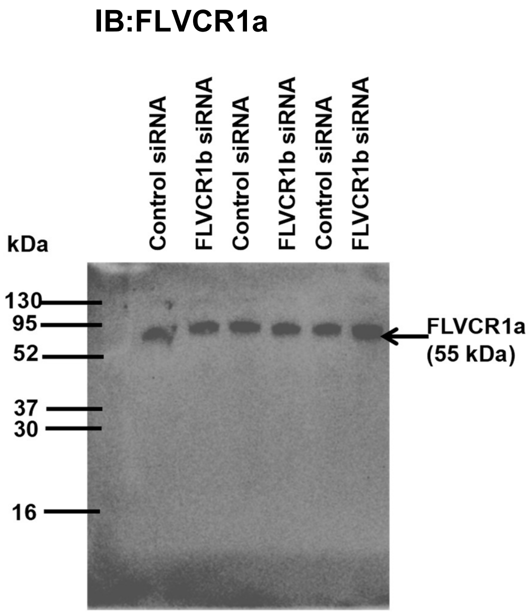

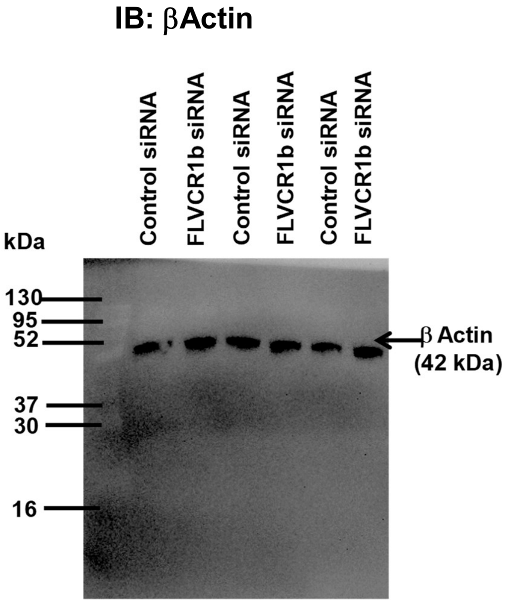

Full western blot scan for Supplementary Figure 1F

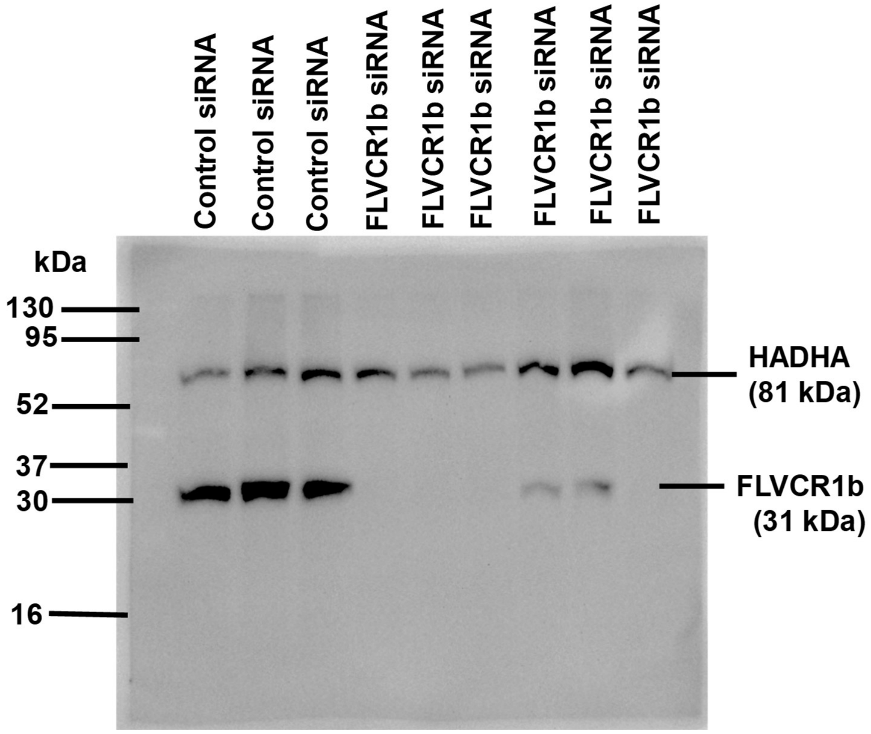

Full western blot scan for Supplementary Figure 2A

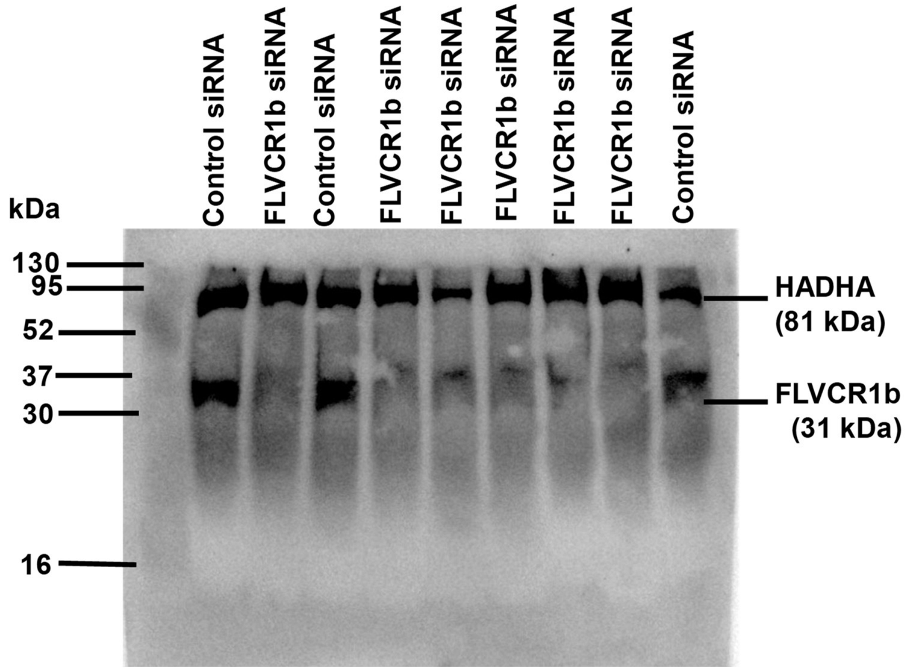

Full western blot scan for Supplementary Figure 2C

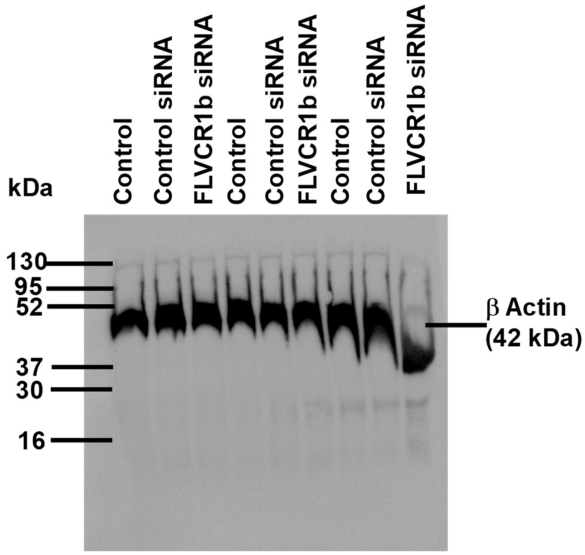

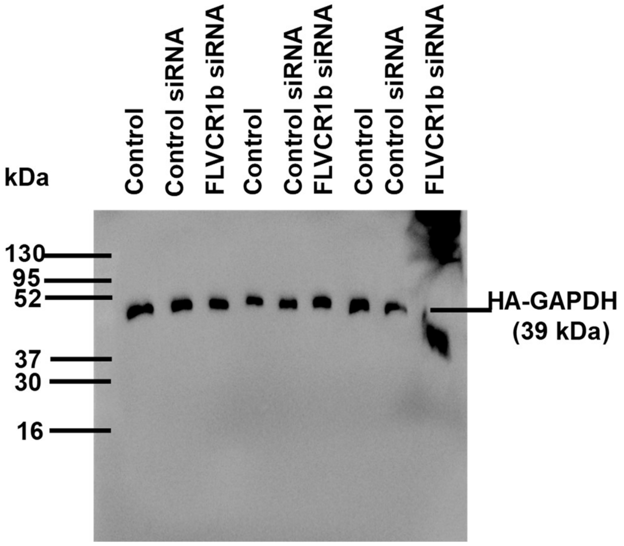

Full western blot scan for Supplementary Figure 3A

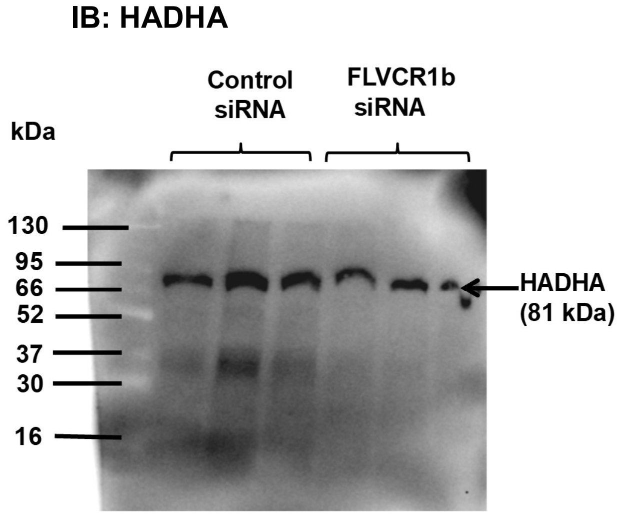

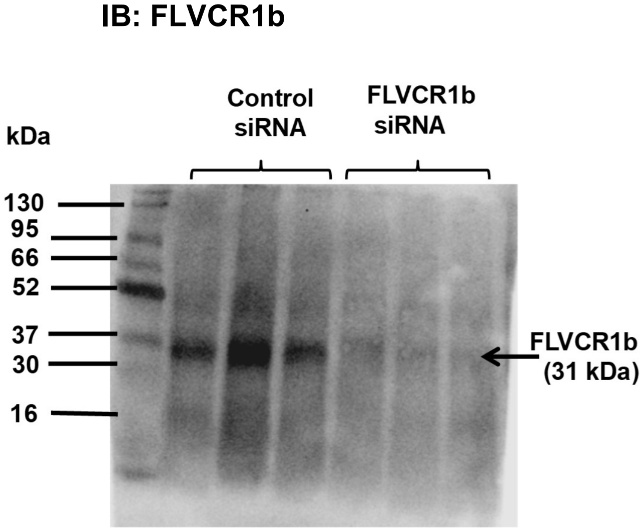

Full western blot scan for Supplementary Figure 3C

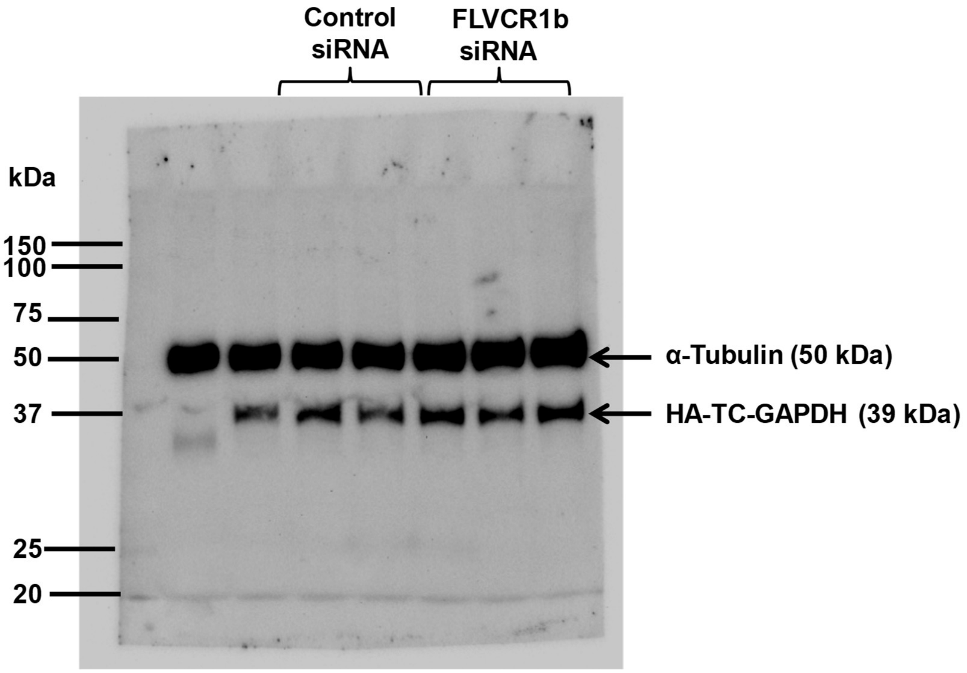

Full western blot scan for Supplementary Figure 4A

IB: IDO1

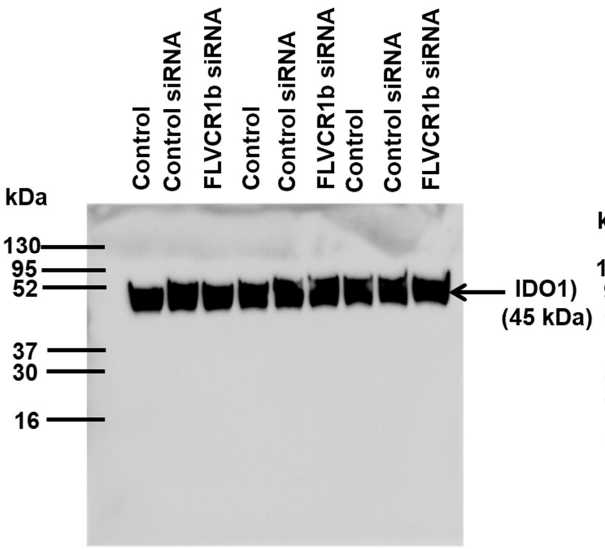

IB: βActin

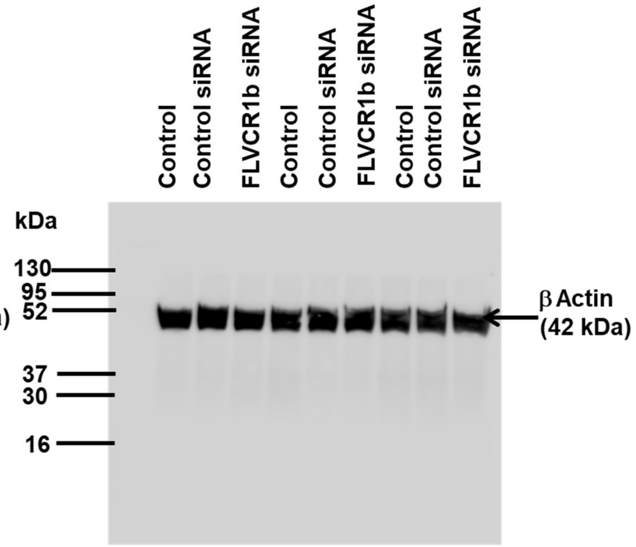

Full western blot scan for Supplementary Figure 4B

IB:sGCβ

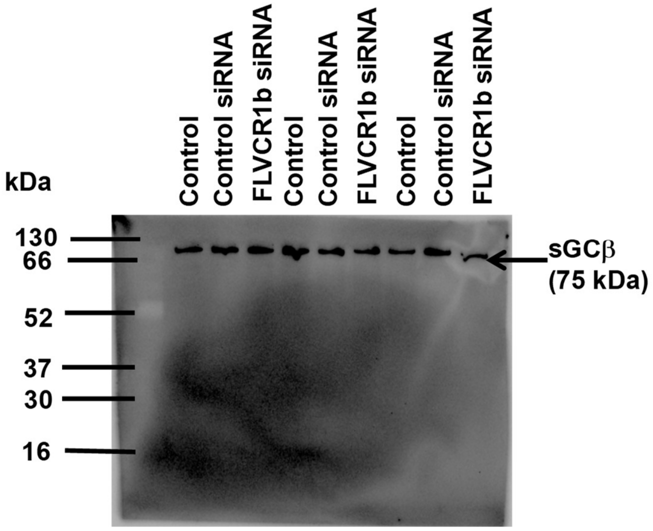

IB: βActin

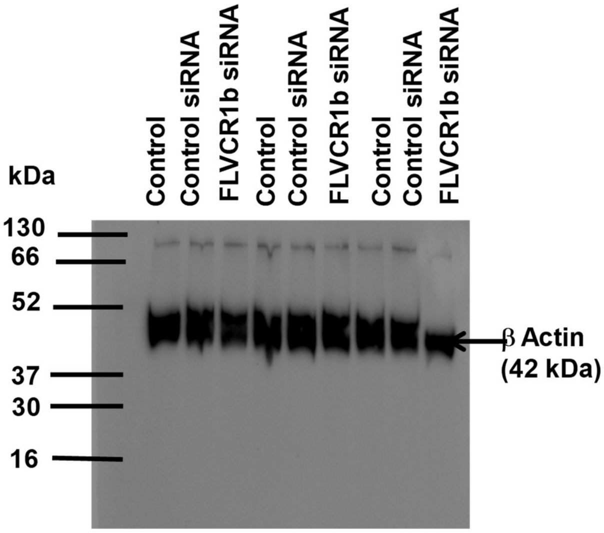

Full western blot scan for Supplementary Figure 5A

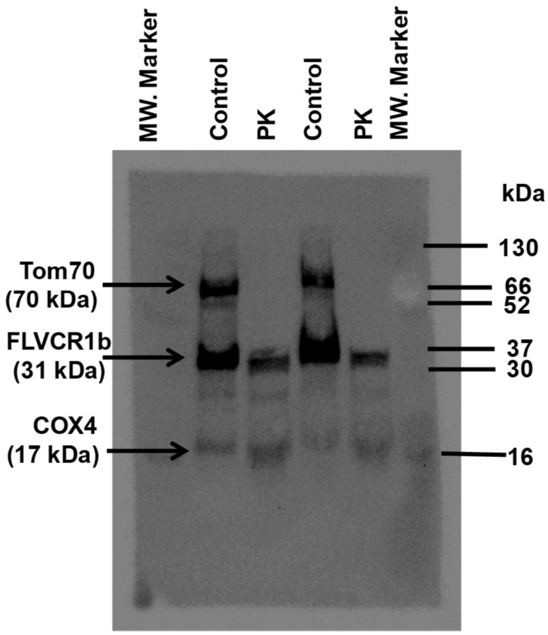

Full western blot scan for Supplementary Figure 5C

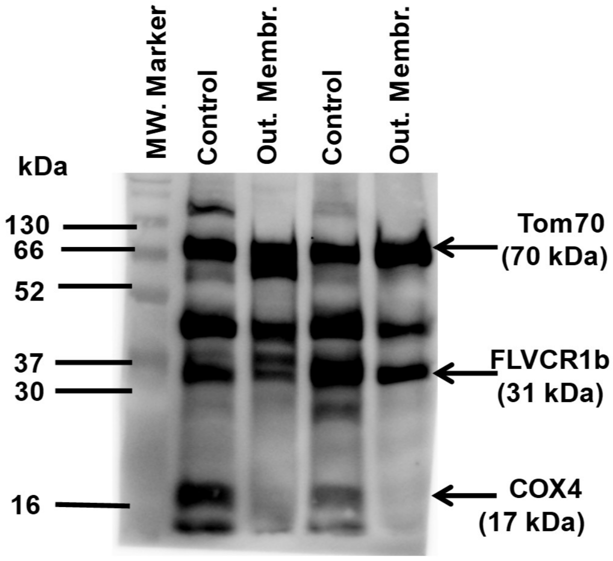

Full western blot scan for Supplementary Figure 5D

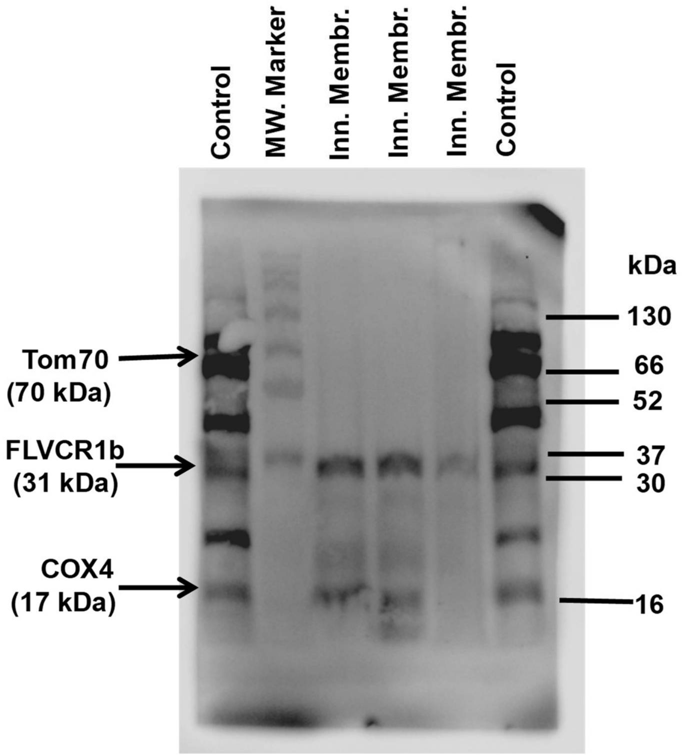

Full western blot scan for Supplementary Figure 9A

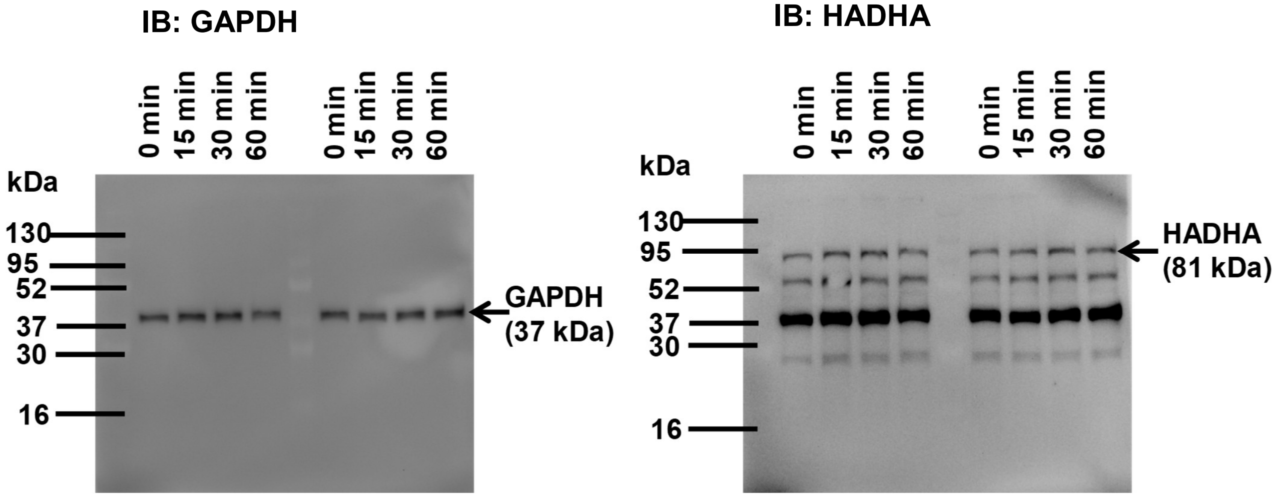

Full western blot scan for Supplementary Figure 9B

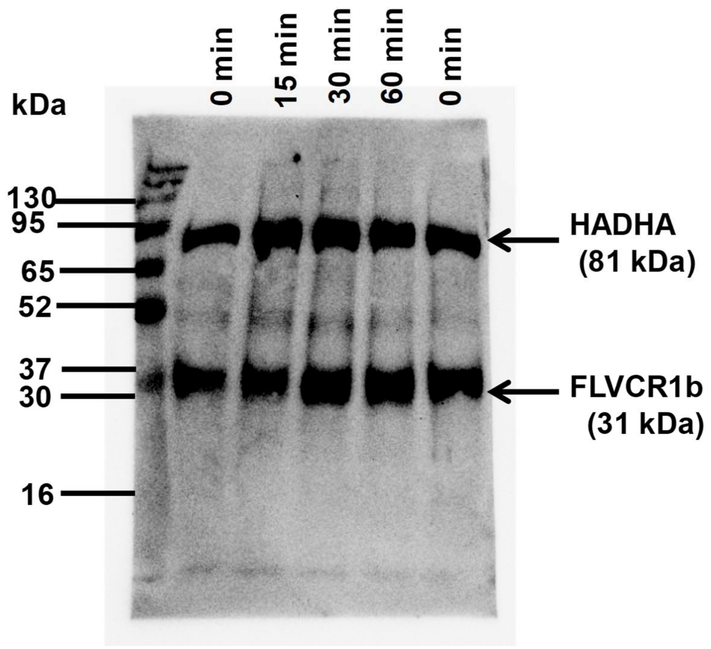

Full western blot scan for Supplementary Figure 9C

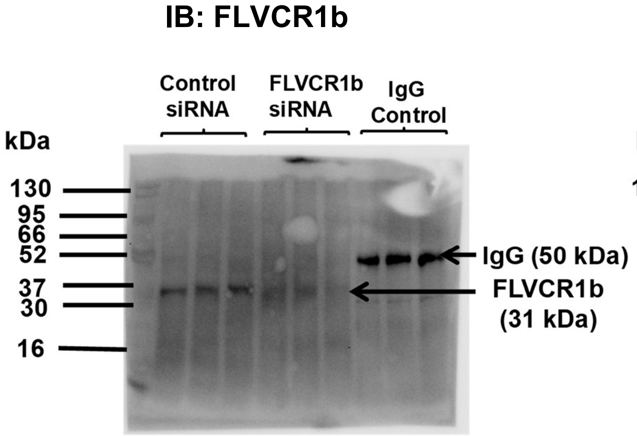

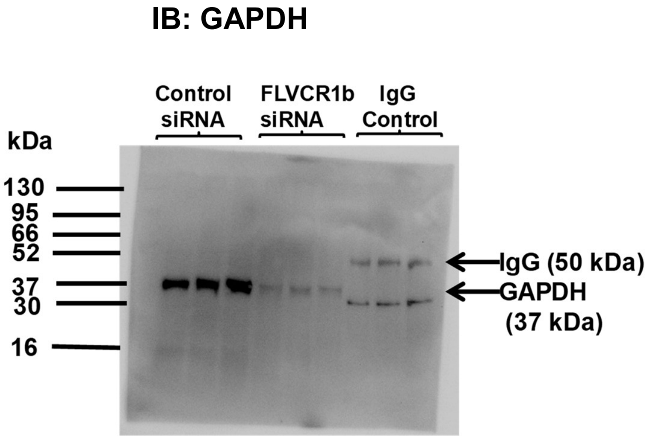

Full western blot scan for Supplementary Figure 11E

IB: FLVCR1b

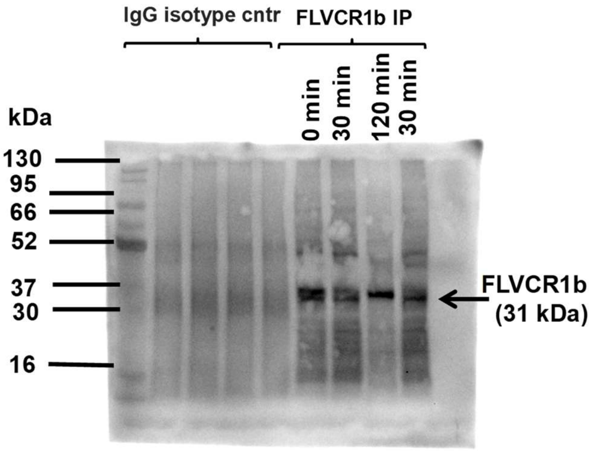

IB: HA

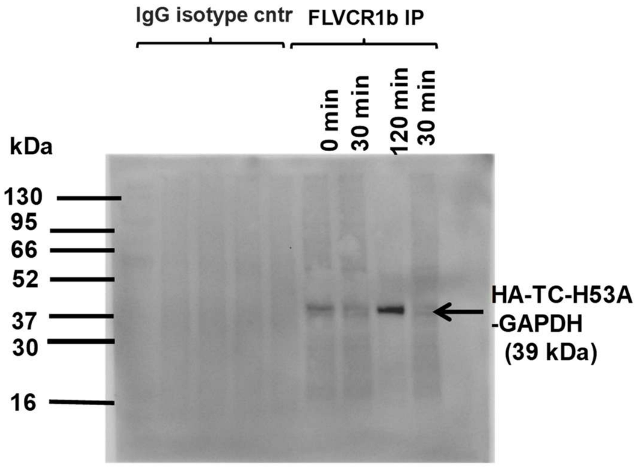

Full western blot scan for Supplementary Figure 11F

IB: Tubulin

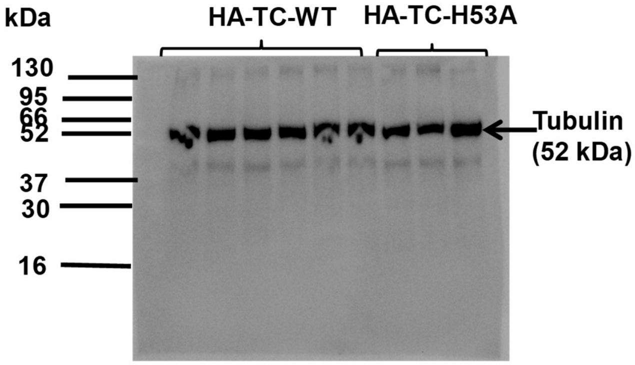

IB: HA

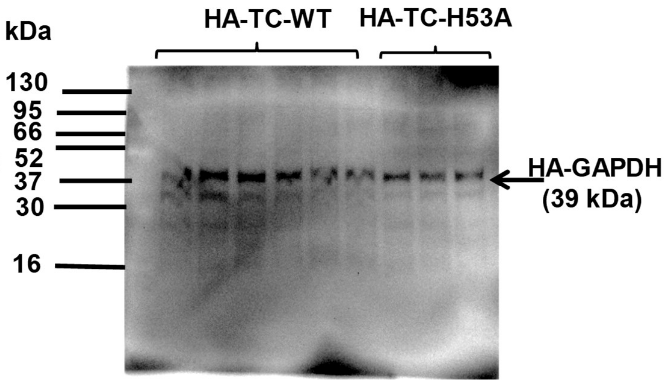

Full western blot scan for Supplementary Figure 12A

IB: HADHA

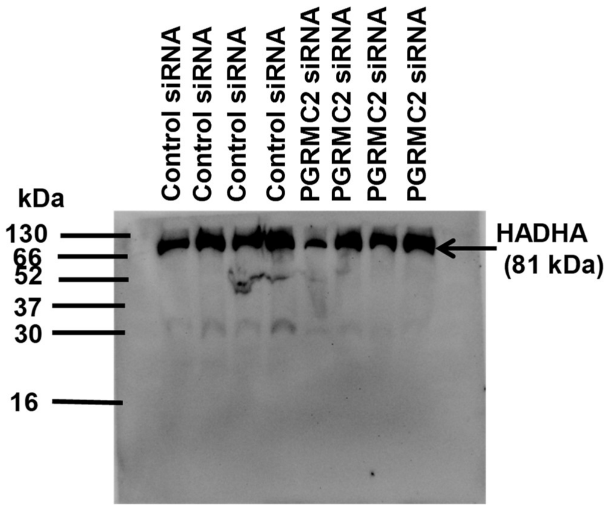

IB: PGRMC2

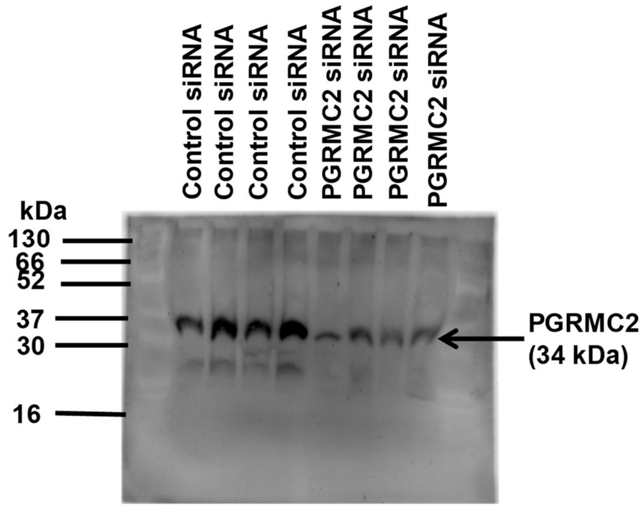

Full western blot scan for Supplementary Figure 12C

IB: βActin

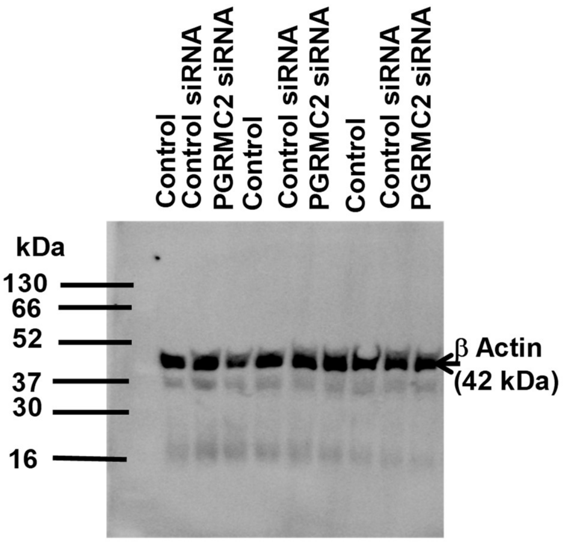

IB: HA

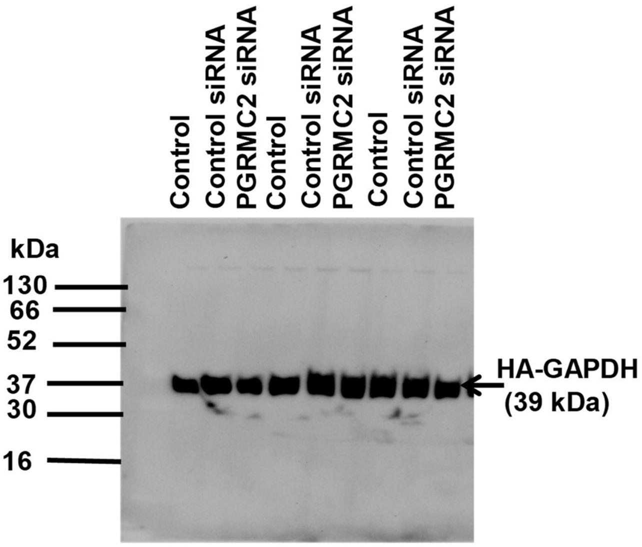

Full western blot scan for Supplementary Figure 12D

IB: IDO1

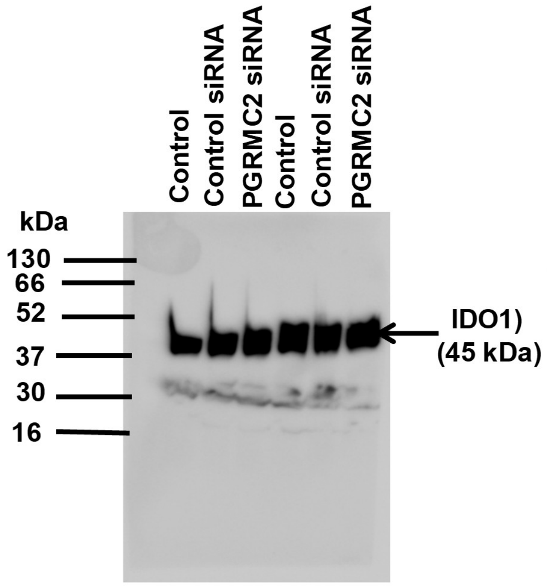

IB: βActin

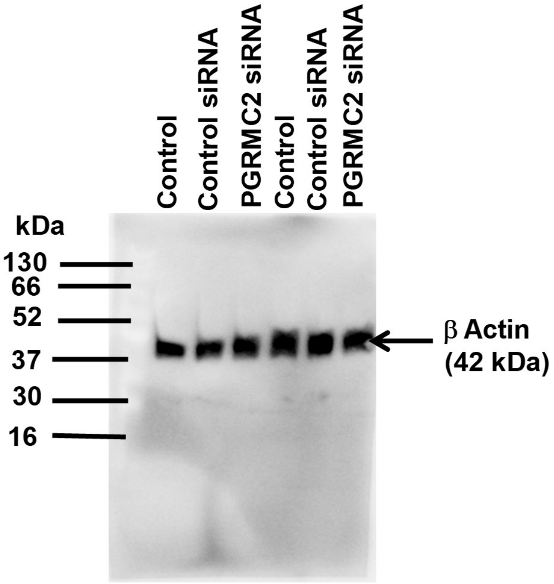

Full western blot scan for Supplementary Figure 12E

IB: sGCβ

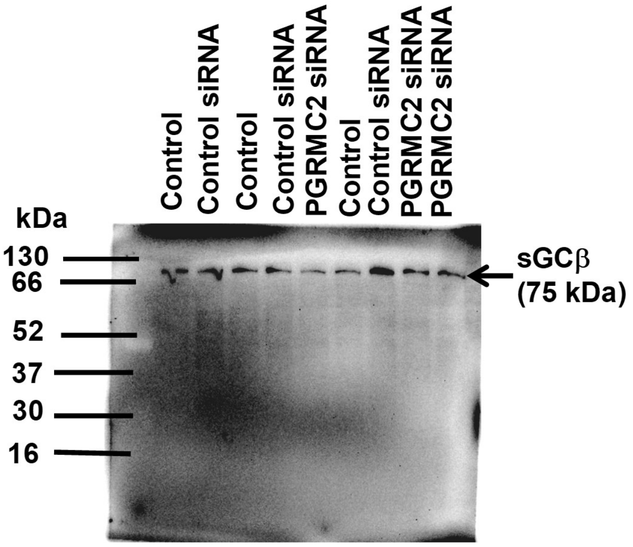

IB: βActin

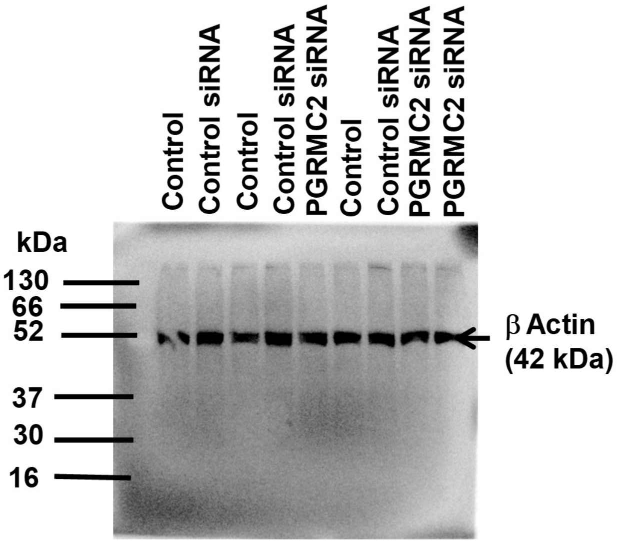

Full western blot scan for Supplementary Figure 14A

IB: βActin

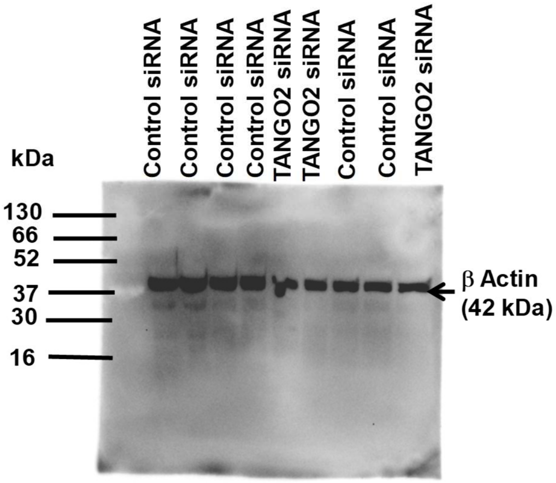

IB: TANGO2

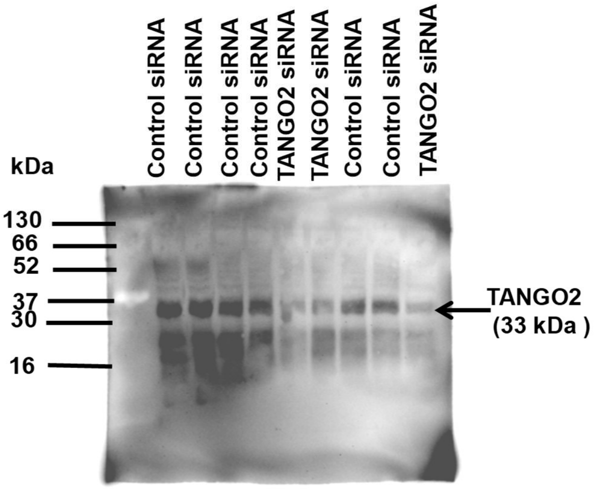

Full western blot scan for Supplementary Figure 14C

IB: βActin

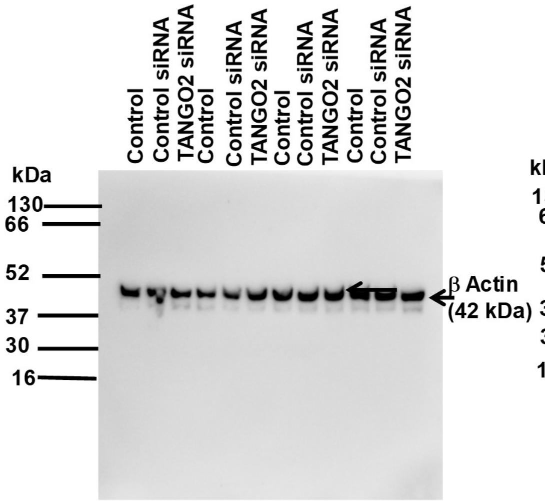

IB: HA

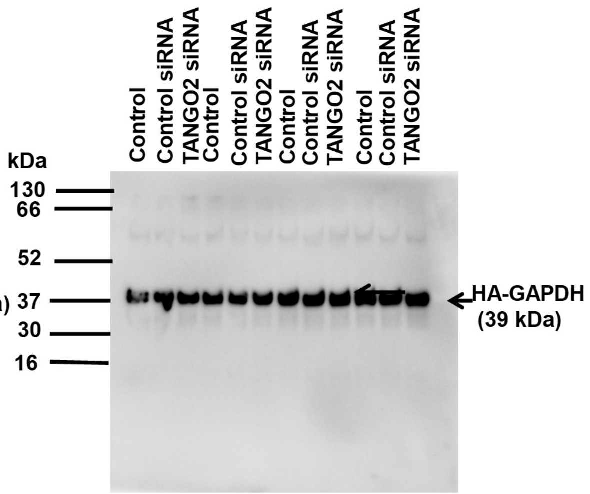

Full western blot scan for Supplementary Figure 14D

IB: IDO1

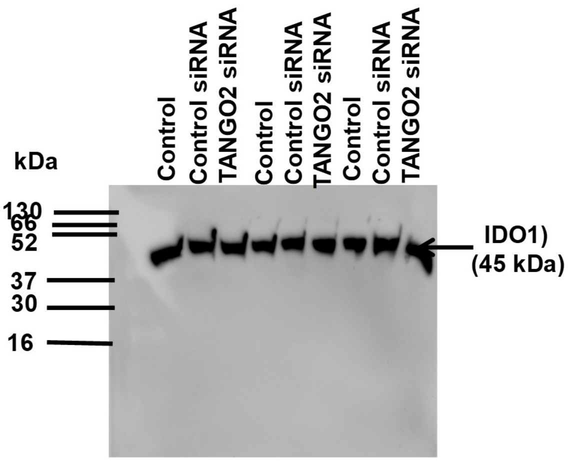

IB: βActin

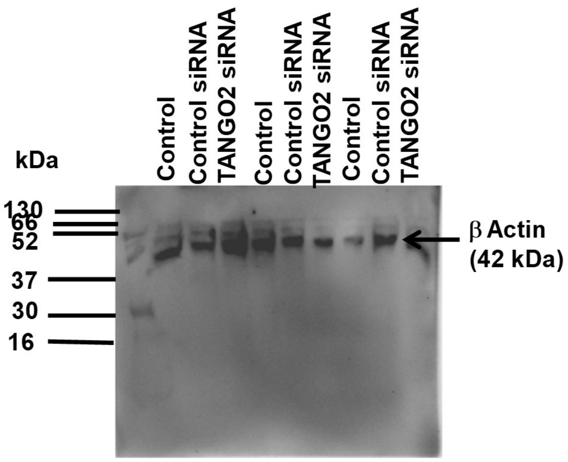

Full western blot scan for Supplementary Figure 14E

IB: sGCβ

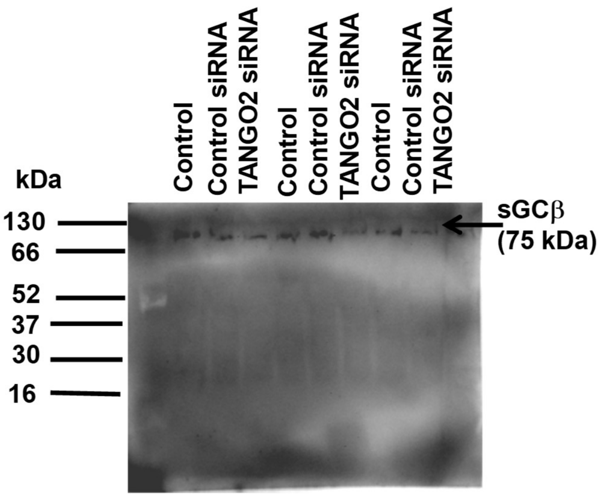

IB: βActin

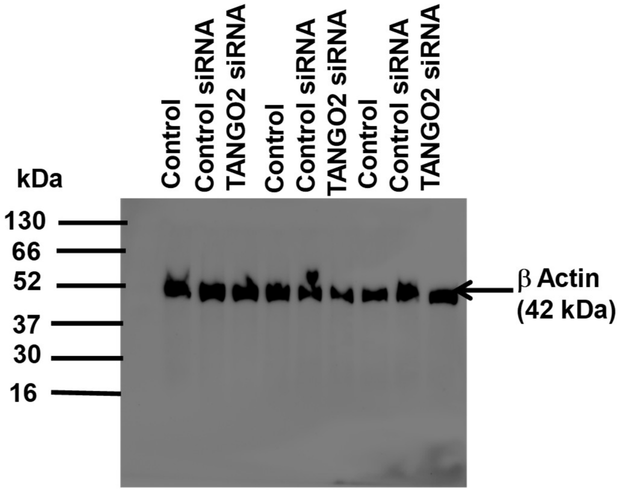

Full western blot scan for Supplementary Figure 15C

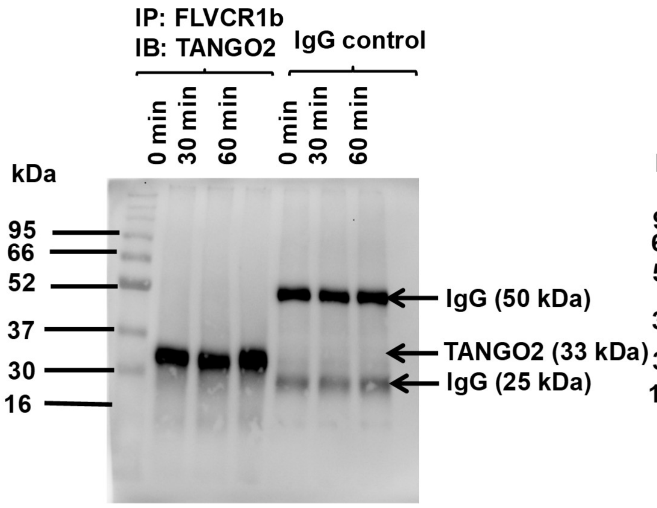

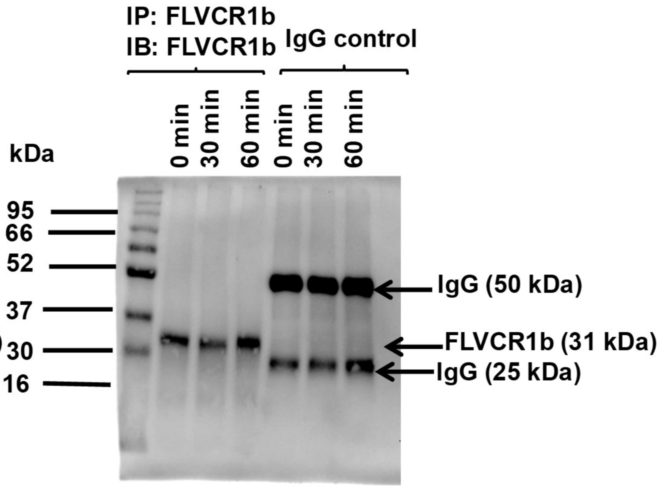

Full western blot scan for Supplementary Figure 15D

IB: TANGO2

IB: HADHA

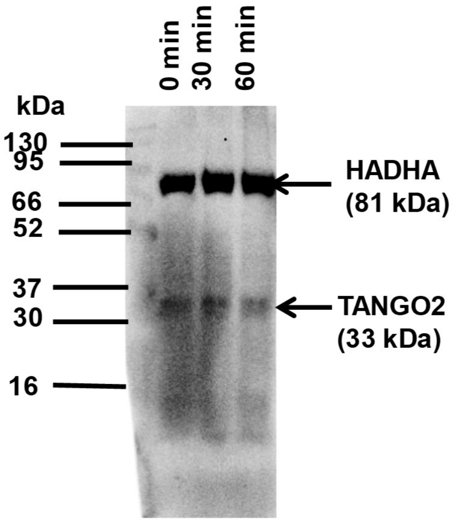

IB: FLVCR1b

IB: HADHA

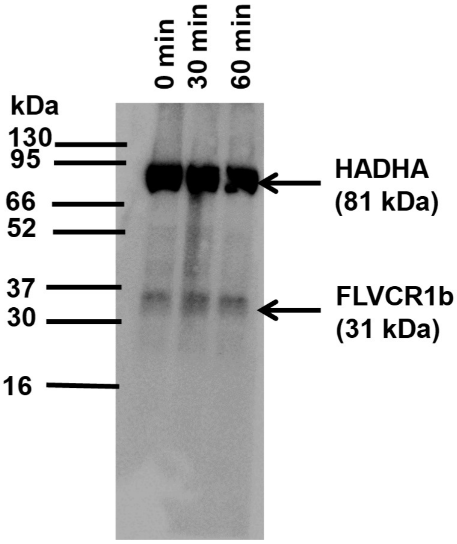

Full western blot scan for Supplementary Figure 16A

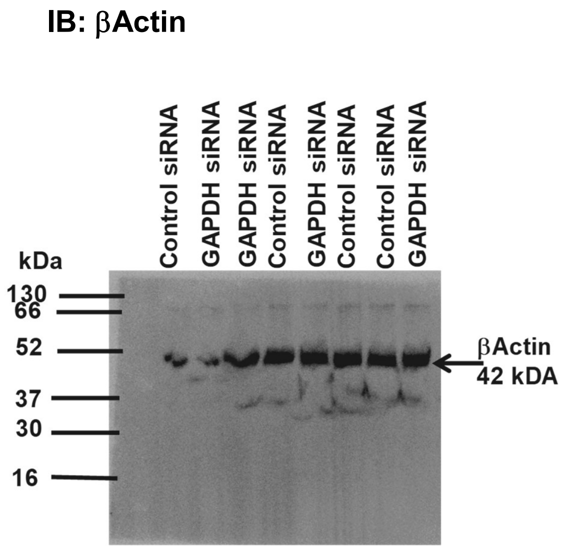

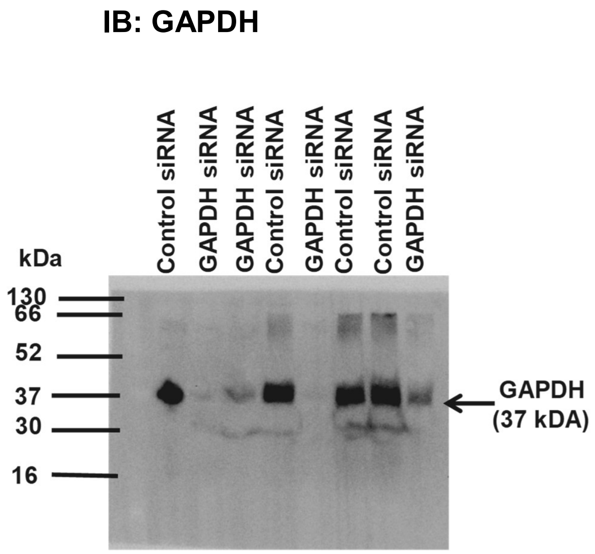

Supplement: Supplementary file 1 — Supplementary Information [file 41467_2025_62819_MOESM1_ESM.pdf]
